# Three-dimensional printing of nanomaterials-based electronics with a metamaterial-inspired near-field electromagnetic structure

**DOI:** 10.1126/sciadv.adz7415

**Published:** 2026-02-06

**Authors:** Jian Teng, Samuel H. Hales, Xin Yang, Jared Anklam, Saebom Lee, Yu Liu, Dwipak Prasad Sahu, Leibin Li, Cordelia Latham, Xi Tian, Derrick Wong, Taylor E. Greenwood, John S. Ho, Yong Lin Kong

**Affiliations:** ^1^ Department of Mechanical Engineering, Rice University, Houston, TX 77005, USA.; ^2^ Rice Advanced Materials Institute, Rice University, Houston, TX 77005, USA.; ^3^ Department of Mechanical Engineering, University of Utah, Salt Lake City, UT 84112, USA.; ^4^ Department of Electrical and Computer Engineering, National University of Singapore, Singapore 117583, Singapore.

## Abstract

Three-dimensional (3D) printing can create freeform architectures and electronics with unprecedented versatility. However, the full potential of electronic 3D printing has so far been limited by the inability to selectively anneal the printed materials, especially on temperature-sensitive substrates. Here, we achieve highly selective and rapid volumetric heating of 3D-printed nanomaterials and polymers in situ by focusing microwaves using a metamaterial-inspired near-field electromagnetic structure (Meta-NFS). In contrast to previous work, the Meta-NFS achieves the spatial resolution and power density needed to 3D print freeform microstructures where the electronic and mechanical properties can be locally programmed even within optically opaque materials. By broadening the material palettes compatible with 3D printing, near-field microwave 3D printing with Meta-NFS enables classes of electronics that are otherwise challenging to create.

## Teaser

Near-field microwaves with Meta-NFS enable selective and rapid in situ heating of 3D-printed nanomaterials.

## INTRODUCTION

The ability to seamlessly interweave functional nanomaterials using three-dimensional (3D) printing (aka. additive manufacturing) enables the creation of complex multimaterial electronics that are unattainable using traditional subtractive manufacturing technologies (*1*–*5*). However, the inability to selectively anneal printed nanomaterials, especially on temperature-sensitive constructs, has limited the functional complexity and performance of 3D-printed devices. Thermal annealing dictates 3D-printed electronic performance by (i) merging otherwise disconnected nanomaterials, (ii) reducing defects and interfaces, (iii) removing additives, and (iv) improving contact between printed layers. Prior works primarily rely on heating the entire printed object in a bulk annealing process (*3*, *6*–*8*), which severely limits the possible material integration and geometrical configurations. For example, the substrate and other integrated materials have to withstand both the annealing temperature and the generated stress due to the thermal expansion mismatch between materials in the annealing process. Alternatively, the potential performance of the printed device is limited by the possible range of annealing temperature or ink formulations with larger (micrometer scale) particles (*9*, *10*) or “self-sintering” additives (*11*, *12*).

Highly focused electromagnetic energy can achieve in situ localized heating of printed material to program the construct’s functional properties, as demonstrated with laser annealing (*13*, *14*). However, the material integration achievable with laser annealing is largely limited to print materials with high optical absorption and substrates with low optical absorption (fig. S1A) (*2*, *15*). In contrast, microwave heating depends on ohmic losses in metal particles with sizes smaller than the skin depth, or on dielectric losses in nonconductive materials (*16*, *17*). This mechanism enables selective and rapid volumetric heating of the print materials on substrates that are incompatible with laser annealing (*16*), such as highly optically absorbing polymers or biological media. Previous studies have used microwaves for postfabrication processing, for example, bonding plastics by placing a carbon nanotube (CNT) sheet at the interface between polymer plates and irradiating the stack (*18*) and welding 3D-printed thermoplastic interfaces through intense localized heating of CNTs (*19*). However, these approaches relied on applying microwaves after fabrication rather than enabling in situ, localized, volumetric annealing during the printing process.

While microwave heating enables selective and rapid volumetric heating, due to the diffraction limit (*20*–*22*), it remains challenging to focus microwaves with longer wavelengths to the spatial resolution and power density required for micrometer-scale 3D printing. Although several near-field applicators have been proposed, some cannot reach the power density necessary for thermal annealing and are only suitable for near-field microscopy because of inefficient electromagnetic field confinement (*23*). Others rely on bulky resonant chambers to enhance the field strength, but the annealing spot size is limited to the millimeter scale due to the low spatial field uniformity of the proposed coaxial applicators (*24*–*27*).

**Fig. 1.**
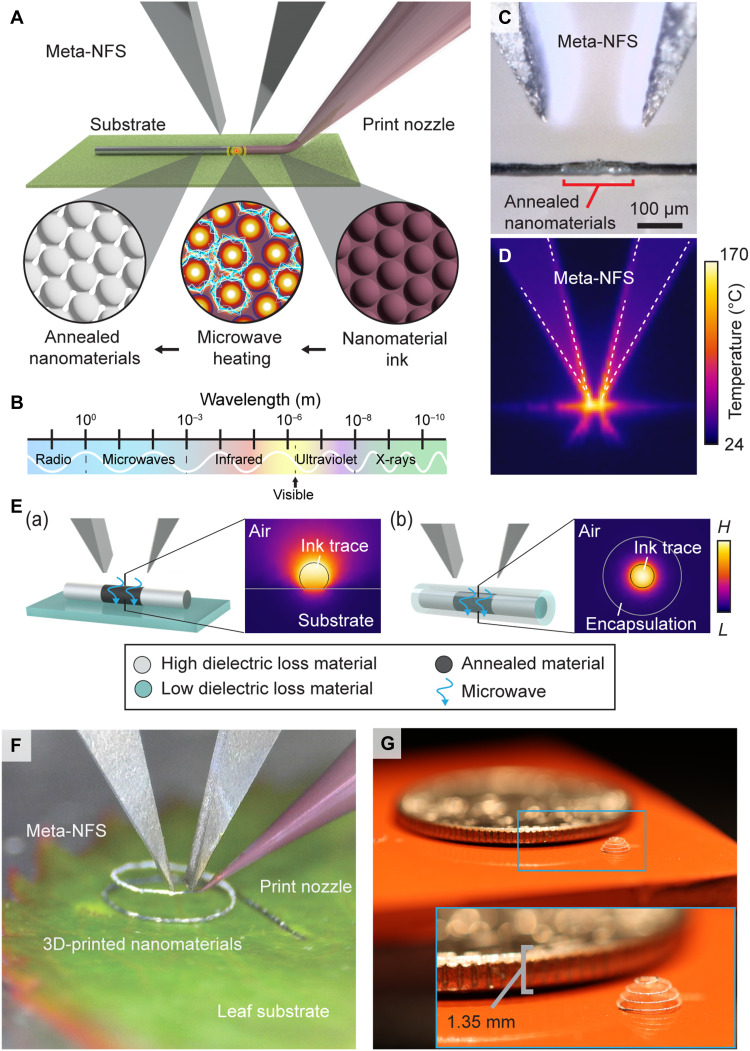
Highly selective and rapid volumetric heating of 3D-printed materials with a Meta-NFS. (**A**) Schematic of the 3D printing process with Meta-NFS, illustrating the in situ selective annealing of 3D-printed materials, which is possible even on a temperature-sensitive substrate. (**B**) Microwave spectrum schematic. (**C**) Optical image and (**D**) IR image of a Meta-NFS during the heating of a submillimeter (~150 μm wide) region of viscoelastic silver-nanoparticle ink on a polyethylene substrate. (**E**) Representative examples showing the Meta-NFS volumetric dielectric heating of inks on low-loss (A) temperature-sensitive substrate, or within (B) multimaterial constructs, even when fully encapsulated. The accompanying heatmaps show the simulated heat distribution induced by the microwave. H and L denote high and low temperatures, respectively. (**F** and **G**) Photographs showing the printing of freestanding silver microarchitecture with a 30-μm trace diameter on a leaf, demonstrating the ability to print on a temperature-sensitive substrate and on silicone, demonstrating the ability to achieve high-resolution features.

Here, we introduce near-field microwave 3D printing (NFP), which leverages a metamaterial-inspired, near-field electromagnetic structure (Meta-NFS) to achieve submillimeter microwave heating of printable inks in situ during a microextrusion-based 3D printing process (Fig. 1A). A split-ring resonator–based structure, inspired by the unit element designs widely used in metamaterials, is used to engineer the distribution of electromagnetic fields (*28*, *29*). During NFP with Meta-NFS, microwaves with 1-mm to 1-m wavelengths are absorbed in nanomaterial inks extruded from a nozzle (Fig. 1B), which enables rapid and volumetric heating with highly focused near-field microwave energy to modulate their mechanical and functional properties. In contrast to earlier work (*24*, *25*), NFP with Meta-NFS can selectively anneal nanomaterial inks with submillimeter resolution (<200 μm, Fig. 1C) to temperatures above 160°C (Fig. 1D) within the ink and provides opportunities for in situ material synthesis (*30*, *31*) during printing. Meta-NFS can achieve in situ annealing via dielectric properties of ink materials rather than optical properties (Fig. 1E). The use of the Meta-NFS allows for the creation of multimaterial constructs even when the materials are completely encapsulated inside a temperature-sensitive polymer.

The Meta-NFS approach is particularly effective for a broad range of functional nanomaterials such as graphene, CNTs, molybdenum disulfide (MoS_2_), ferrites, and others due to their high microwave absorption (fig. S1B). For instance, graphene can achieve up to 50% absorption of microwave energy compared to only 2.3% absorption of infrared (IR) laser (*32*). Meta-NFS enables selective heating of these critical nanomaterials to create planar or freestanding architectures even on substrates with high optical absorption (e.g., opaque silicone and leaf), as shown in Fig. 1, F and G. By leveraging the dielectric properties of nanomaterials, there is a broad range of circumstances where Meta-NFS provides better selectivity during heating than the conventional annealing methods (table S1).

## RESULTS

### Design of a Meta-NFS for 3D printing

**Fig. 2.**
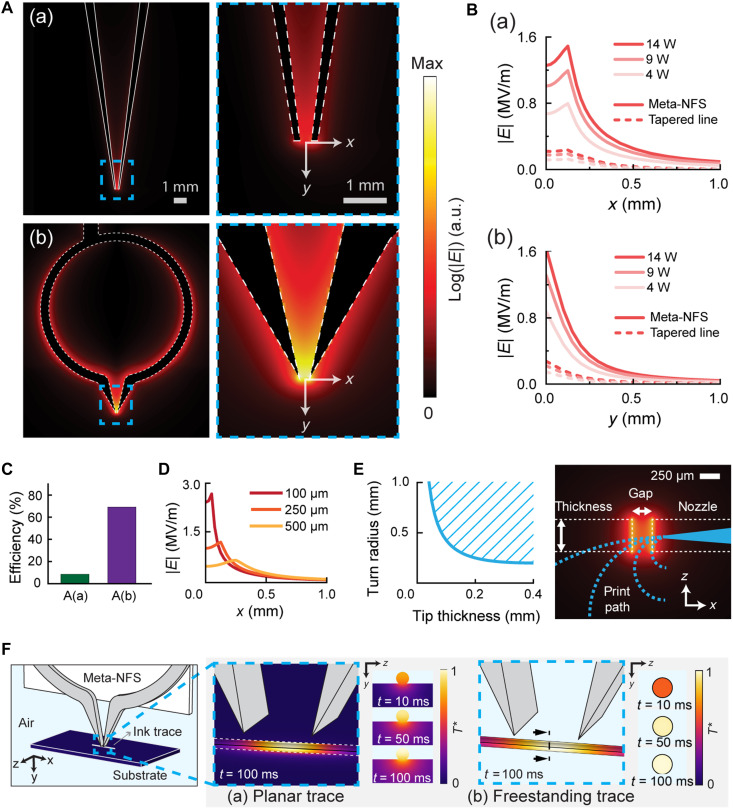
Meta-NFS design and simulation. (**A**) Simulations comparing the electric-field *E* magnitude of the Meta-NFS (b) with the tapered transmission lines conventional microwave structure (a). (**B**) Simulated *E* magnitude with respect to distance from the tip of each structure (a and b) in the *x* and *y* directions, where *x* indicates the spot size and *y* is the working distance. The *x*-direction plots are taken at *y* = 50 μm, where ink is positioned. (**C**) The efficiency of microwave power transfer to the tip for the designs in A(a) and A(b). (**D**) The simulated *E* decay from the Meta-NFS tip with different gap sizes between the tips (100 to 500 μm). (**E**) Calculated achievable turn radius (left) of the printing setup with respect to Meta-NFS tip thickness. Image (right) shows the potential print radius overlayed on the simulated top-down *E* magnitude of design A(b). (**F**) Simulated selective heating in a planar silver ink trace on a polyethylene substrate and in a freestanding trace in midair. $T^{*}$ denotes the normalized temperature. a.u., arbitrary unit.

We use a simulation-guided approach to design an electromagnetic structure with the considerations of submillimeter spot size, excitation efficiency, working distance, and electrical field strength. The electromagnetic field decays approximately exponentially with distance, limiting effective coupling beyond the near-field region. A working distance of within 1 mm was therefore chosen to ensure strong, localized energy transfer. This distance is smaller than the Fraunhofer distance (the boundary of near field and far field), defined as $\frac{2D^{2}}{\lambda }$ , where *D* is the largest dimension of the structure and λ is the wavelength (*33*). We started with the conventional transmission line structure [Fig. 2A (a)] (*34*), which can achieve a submillimeter spot size, demonstrating a simulated full width at half maximum of 0.54 mm at 14 W along the *x* axis [Fig. 2B (a)]. However, its low excitation efficiency of 8.5%, resulting from an impedance mismatch between the probe and power source, limits its energy efficiency for achieving the required field strength and its effective working distance, with a maximum electrical field strength of only 0.23 MV/m at 14 W.

To address this limitation, we synergistically integrate a split ring resonator (SRR) (*35*) and tapered tip of traditional near-field focusing structures (*34*) in our probe design [Fig. 2, A and B (b)], which substantially improves impedance matching and enhances excitation efficiency to 79.5% (Fig. 2C), thereby achieving a maximum electrical field strength of 1.49 MV/m at 14 W. The Meta-NFS enables spot size modulation by adjusting tip spacing (Fig. 2D) and provides better field confinement than a standalone SRR structure (fig. S2, A and B).

To minimize substrate-induced dielectric losses, we used a freestanding, substrate-free architecture for the Meta-NFS tips. Tungsten was selected as the tip material for its mechanical stability at high temperatures, which keeps the probe geometry reliable and ensures a consistent resonant frequency for efficient energy transfer. In addition, to reduce heat dissipation to the surrounding substrate on the Meta-NFS during the printing process, we implemented a pulsed excitation mode (with pulse durations of 3 to 100 ms at 0.3 to 10% duty cycle) rather than continuous operation. We note that our Meta-NFS design achieves highly concentrated electrical fields at the Meta-NFS tips, while magnetic fields are negligible (fig. S2C).

The designed Meta-NFS can be integrated with a microextrusion-based 3D printing approach (such as direct-ink writing, as shown in fig. S3) to create freeform microarchitectures where the mechanical and electrical properties can be locally programmed. For example, we demonstrate 3D printing of curvilinear structures by aligning the printed trace to the focused microwaves of the Meta-NFS. In this configuration, the minimum print radius of the architecture is determined by the conductor thickness of the Meta-NFS and the relative distance between the Meta-NFS and the print nozzle (Fig. 2E).

A key advantage of NFP with Meta-NFS is its ability to achieve selective volumetric heating. In microwave heating, the absorbed microwave power in the material is converted into heat, which leads to volumetric annealing in the case of printed ink. A detailed explanation of the microwave heating mechanism is presented in the Supplementary Text. To demonstrate the heat transfer mechanism of the NFP process, we performed numerical simulations. Figure 2F shows the simulation results illustrating the selective heating of planar and freestanding traces. A submillimeter (100 to 200 μm wide) region is primarily heated by the absorbed electric field in the material. The substrate acts as a heat sink, dissipating heat away from the trace through thermal conduction over time. As a result, without the heat sink effect of a substrate, the freestanding trace printed in midair is expected to experience more intense and uniform heating compared to a planar trace. In fig. S4, we further demonstrate the energy absorption and heat transfer at different annealing regions.

### NFP with Meta-NFS

NFP with Meta-NFS enables the printing of freeform microarchitecture, including metal microarchitecture, even on temperature-sensitive substrates. Although bulk metal is known to reflect microwave radiation at room temperature, intriguingly, prior literature has shown that micrometer- and nanometer-scale metal particles heat rapidly when exposed to microwaves (*36*) when their size is on the same scale as the skin depth (*16*). Further, unlike microwave-absorbing dielectric materials, which are heated primarily in the microwave electric field (*16*), metal particles can also be heated in the microwave magnetic field due to multiple effects, such as magnetically induced eddy currents and plasma discharge (*37*, *38*).

**Fig. 3.**
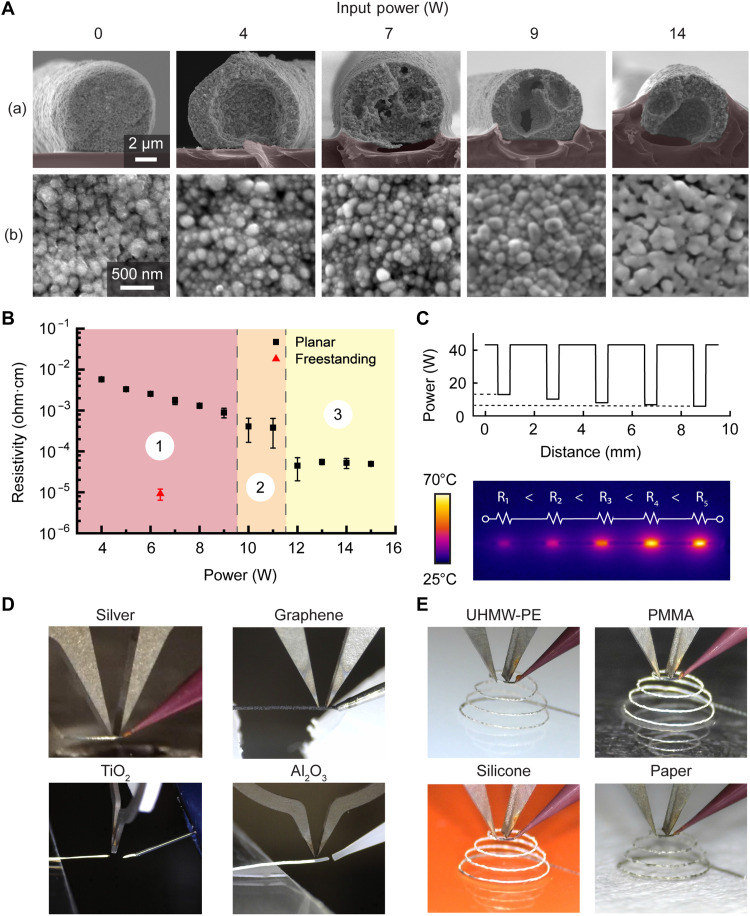
Meta-NFS creates freeform microstructures where the electronic and mechanical properties can be locally programmed and is compatible with a broad range of substrates and nanomaterials. (**A**) SEM images of printed silver nanomaterials with varying Meta-NFS power input, showing the (a) cross section and (b) microstructure of printed specimens. The polymeric substrate (PMMA) is highlighted in red. (**B**) Programmable resistivity of printed traces by modulation of the microwave input power. Plot shows three annealing regions: (1) below, (2) transitioning to, and (3) above dielectric breakdown. Error bars denote the SD of the resistivity measurements from *n* = 3 experiments. (**C**) IR image showing joule heating of a silver trace programmed with six levels of electrical resistivity (resistor length ~600 μm). (**D**) Photographs of freestanding traces printed using diverse nanomaterials. (**E**) Photographs of freestanding microarchitecture with 30-μm trace diameter printed on a broad range of temperature-sensitive substrates.

As a proof of concept, we demonstrate the selective annealing of a silver nanomaterial ink on polymeric substrates such as polymethyl methacrylate (PMMA, glass transition temperature, *T*_g_ ~100°C). Metallic microarchitecture typically requires bulk heating (e.g., 250°C for ~30 min) that would otherwise be incompatible with the *T*_g_ of the substrate (*8*). Owing to the volumetric and selective heating of the high–microwave absorbing metallic ink on a low absorbing [loss tangent, tan(δ) = 0.02 @ 2 GHz] (*39*) polymeric substrate, highly electrically conductive microarchitecture can be printed and annealed with a Meta-NFS, as characterized by the heat-affected zone (HAZ) in Fig. 3A. After annealing, the silver nanoparticle ink has shown 35% expansion with 18% SD (fig. S5), which can be accounted for during the printing process by adjusting the extrusion parameters to produce smaller-diameter traces. The minimum feature size for a single trace is determined by the extrusion nozzle diameter, which depends on the ink properties, such as ink particle size, rheology, and extrusion pressure. The minimum spacing that can be reliably achieved with our current printing setup with Meta-NFS (200-μm tip spacing) using a 30-μm diameter nozzle is 34 μm, as shown in fig. S6.

The electric field concentrated by the Meta-NFS can be increased to exceed the dielectric strength of air (3 MV/m @ 1 atm) (*40*), generating microwave plasma, which subsequently releases thermal energy due to various heating effects, including frictional (ohmic) heating of the energized particles (*40*–*42*). Unlike traditional plasma annealing methods that transfer a plasma to the target material using a flow of feed gas (*41*, *43*), during NFP with Meta-NFS, the target material is positioned within the field of view of the Meta-NFS and will be directly annealed by the focused microwave electric field, generated plasma, and the thermal energy released during plasma formation. When microwave plasma is generated, the HAZ increases [Fig. 3A (a)], as expected, because of the additional contribution of nonselective heating. Such effects could be desirable in several applications (e.g., with thermoplastic polymer) where the trace can be partially engulfed into the substrate. The increased heat can also result in improved annealing of the printed microstructure, reducing the electrical resistivity to the order of 10^−5^ ohm·cm. The freestanding structure shows the best resistivity of 9.2 × 10^−6^ ohm·cm (Fig. 3B and fig. S7, A and B), which is much lower than that of the planar trace annealed with the same microwave parameters (2.9 × 10^−4^ ohm·cm).

Further, we demonstrate the ability to tune material properties in situ on temperature-sensitive substrates by modulating input microwave and printing parameters. Specifically, during NFP with Meta-NFS, increasing the microwave power enhances the densification and grain growth of silver nanoparticle ink, resulting in a decrease of electrical resistivity by over three orders of magnitude (down to 4.9 × 10^−5^ ohm·cm at 15 W, approaching the conductivity of bulk silver (1.6 × 10^−6^ ohm·cm) (Fig. 3B). We performed tensile tests of five printed silver traces, which show an average Young’s modulus of 945 MPa with an SD of 116 MPa (fig. S8A). The printed silver trace’s strength is sufficient to support a suspended screw weighing 1.7 g, as shown in fig. S9 and movie S7. In addition, the HAZ can be reduced by optimizing the power and pulse width combination (fig. S7C). Therefore, by modulating the input power, our approach can achieve in situ tuning of trace resistivity along a single filament, as demonstrated with variations in IR radiation generated from the joule heating of programmed resistive elements when a direct electrical current is applied (8 mA, Fig. 3C). In most cases, achieving such seamless transition of material properties in a single print material is challenging with conventional bulk annealing and multimaterial printing approaches. We note that electrical resistivity increases with increasing trace width, due to the increased volume being heated by the microwave. However, this can be finely tuned by adjusting the input power to achieve the desired resistivity (fig. S10).

To demonstrate the general material compatibility of our NFP with Meta-NFS, we print micro-traces using diverse conductive, semiconductive, and dielectric materials, as shown in Fig. 3D; figs. S11, C and D and S12; and movie S3. In addition to nanomaterials, we demonstrated that NFP can also 3D print thermoset epoxy–based microarchitecture. The Meta-NFS enables in situ rapid cross-linking of an epoxy ink containing <5 wt % additives (CNTs and a rheological modifier) to improve microwave absorption. As a proof of concept, we also printed a freestanding microspring that could withstand >250% elastic strain with a spring constant of 0.271 N/m (fig. S11, A and B). This in situ annealing capability, applicable to a broad range of materials, unlocks the creation of more complex freeform geometries compared to previous methods that rely solely on tuning rheological properties (*8*, *44*). Therefore, it holds strong potential for bioelectronic applications, such as integrating actuating mechanisms or cantilevers for sensing onto temperature-sensitive biomedical devices and materials (*45*, *46*).

**Fig. 4.**
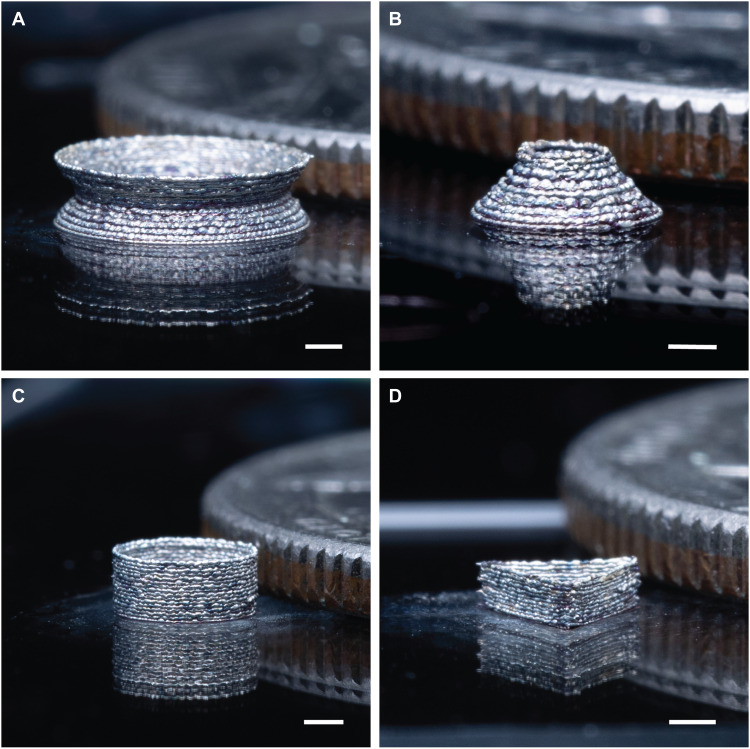
Photograph of 3D architectures printed by the layer-by-layer deposition approach using Meta-NFS. (**A**) Silver biconical structure. (**B**) Silver frustum. (**C**) Silver ring. (**D**) Silver triangular prism. The architectures were placed next to a US quarter coin (diameter, 24.26 mm; thickness, 1.75 mm). Scale bar, 1 mm.

We further demonstrate the selective heating and freeform structuring ability of NFP with Meta-NFS on a range of temperature-sensitive substrates that are important in the creation of a broad range of flexible, stretchable, and biodegradable electronics (*47*–*49*). Specifically, we show the ability to anneal silver nanoparticle traces to a resistivity below 8 × 10^−3^ ohm·cm at 7 W input power on substrates such as PMMA, silicone, ultrahigh–molecular weight polyethylene (UHMW-PE, *T*_m_ ~ 150° to 160°C) (*8*), and paper, which is otherwise challenging to integrate (Fig. 3E). The microspirals are mechanically robust and can experience 70% elastic strain, with a measured spring constant of 7.8 N/m (fig. S13, movie S1, and fig. S8B). The unique capability to create such microstructures in 3D freestanding configurations expands the possible device design and multifunctional integration in comparison with traditional planar devices or previous works in freeform printing that require chemical additives (*50*–*52*). For example, 3D microinductors and antennas with higher efficiency can be created when they are no longer limited to planar configurations (*53*, *54*). In addition, our approach is not limited to wireframe architectures; by adopting a layer-by-layer approach, Meta-NFS can fabricate complex 3D architectures, as demonstrated in Fig. 4.

**Fig. 5.**
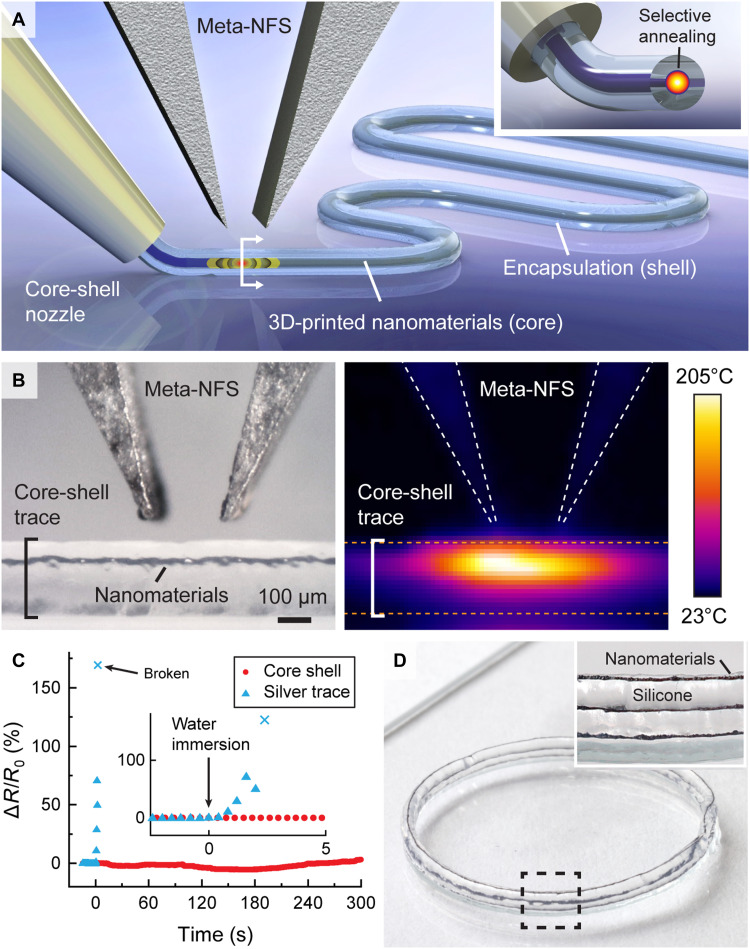
Selective annealing enables microscale 3D printing within a fully encapsulated structure. (**A**) Schematic demonstrating multimaterial printing with a core-shell nozzle, where the embedded nanomaterials at the core are selectively annealed in situ inside a polymeric shell during the printing process. (**B**) Optical image (left) and IR image (right) showing NFP annealing of a core-shell trace consisting of silver nanomaterials embedded within silicone elastomer. (**C**) Relative change in resistance of a core-shell trace and unembedded silver nanomaterials when immersed in water. (**D**) Photographs of a cylindrical inductor printed using core-shell NFP, showing local and selective programming of the target materials at the core (silver nanomaterials, 30-μm trace) without visible damage to the shell encapsulating the ink.

By adjusting microwave and printing parameters (e.g., resonant frequency, input power, pulse width, and extrusion pressure), the material selectivity of NFP with Meta-NFS also allows for the selective microwave annealing of nanomaterials, even when the nanomaterials are fully encapsulated by a low-absorption or optically opaque polymer. Such capability can enable seamless multimaterial integration of freeform electronics with polymeric encapsulants, not possible with traditional annealing approaches (e.g., laser, oven, or plasma annealing). As a proof of concept, we printed core-shell traces using NFP with Meta-NFS (Fig. 5A), consisting of a silver nanomaterial core and a silicone elastomer shell [tan(δ) = 0.023 @ 2 GHz] (*55*). To demonstrate the material selectivity of this approach, we compared the microwave absorption of a core-shell trace and an experimental control by heating both with a Meta-NFS and observing the resulting temperature increase.

As shown in Fig. 5B, the nanomaterials embedded as the core of a multilayer trace can be selectively heated to >200°C, in contrast with the experimental control, pure silicone that does not contain embedded silver nanomaterials, which is only heated to ~65°C under the same microwave power (fig. S14A). The focused annealing of NFP coupled with its material selectivity enables in situ tuning of the properties of the target material. For example, we show that the silver core resistivity can be programmed by modulating input microwave power even when it is fully embedded inside the silicone polymer (fig. S14B, IR radiation from the Joule heating of the programmed resistive element). The ability to create and tune multimaterial traces enables the incorporation of multifunctionalities in complex structures, such as the multilayer fabrication of devices with a conductive core and an insulating shell. In this particular example, silicone enables seamless insulation of the silver nanomaterials from exposure to solvents such as water, where the electrical conductivity (5.6 × 10^−2^ ohm·cm) is robustly maintained with core-shell silicone for over 300 s in contrast to the rapid dissolution of an unencapsulated trace in ~2.5 s (Fig. 5C). To further demonstrate the ability to create multimaterial devices, we print a continuous, multilayer cylindrical inductor coil using core-shell NFP with Meta-NFS (Fig. 5D and movie S2). The inductor coil achieves a quality factor (Q-factor) of 6.5 at its wirelessly interrogated self-resonant frequency of 1.12 GHz.

### Functional devices

**Fig. 6.**
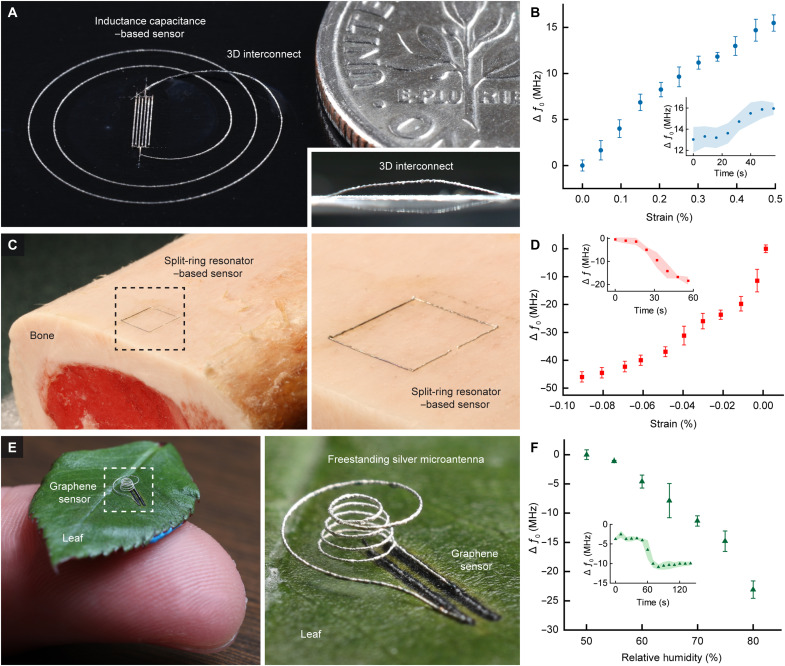
Multimaterial printing of hybrid microdevices. (**A**) Microsensor printed on polyethylene, relative to a US dime (dime diameter, 17.91 mm; thickness, 1.35 mm). (**B**) The wirelessly measured Δ*f* of the microsensor (A) with respect to changes in measured mechanical strain. The total 0.5% strain corresponds to ~50 μm of displacement across the sensor. Inset shows rapid transient response of the Δ*f* during an increase from 0.4 to 0.5% strain. The results are representative of three measurement trials; each error bar denotes the SD of ~200 measurement points in the representative trial. (**C**) Metamaterial-inspired microsensor printed on a bovine femur bone (sensor side length, 4.5 mm). (**D**) The wirelessly measured Δ*f* of the microsensor (C) with respect to changes in measured mechanical strain. The total 0.1% strain corresponds to ~5 μm of displacement across the sensor. Inset shows rapid transient response of the Δ*f* during a decrease from 0 to 0.01% strain. The results are representative of three measurement trials; each error bar denotes the SD of ~200 measurement points in the representative trial. (**E**) Photographs showing a wireless humidity sensor printed on a leaf using NFP, consisting of a freestanding silver microantenna (30-μm trace diameter) connected to a graphene capacitive sensor. (**F**) The wirelessly measured change in resonant frequency Δ*f* of the sensor (E) in response to changes in RH. Inset shows the measured Δ*f* during an increase from 60 to 65% RH at 24°C. Error bars denote the SD of the measurements from *n* = 3 experiments.

As a proof of concept, we leverage the selective annealing approach of NFP with Meta-NFS to print wireless strain sensors on biocompatible polymers, such as UHMW-PE, and biological constructs, such as bovine femur bone. A fabricated sensor on UHMW-PE (Fig. 6, A and B) achieves an average sensitivity of 30.9 MHz/% strain (root mean squared error: 0.98) and a Q-factor of 3.2 at its resonant frequency *f_0_* of 1.17 GHz. The ability of NFP with Meta-NFS to directly print functional nanomaterials onto polymeric substrates such as UHMW-PE, which is used in biomedical implants (e.g., total-joint replacement arthroplasties) due to its chemical stability, could enable wireless strain monitoring of an implanted device without modifying the original structure of the implant. NFP with Meta-NFS also enables a freestanding connection between the inductor and capacitor, which increases device functional density with a single manufacturing step in comparison with conventional multilayer lithography processes (Fig. 6A and movie S4). Similarly, the ability of NFP with Meta-NFS to directly print wireless sensors onto biological constructs, such as bone, could enable deep tissue remote sensing for a broad range of biomedical diagnoses (*21*). As a demonstration, we printed a wireless strain sensor based on the split-ring resonator metamaterial on a bovine femur bone (Fig. 6, C and D). The fabricated sensor achieves an average sensitivity of 616.6 MHz/% strain (root mean squared error: 0.94) and a Q-factor of 27.9 at its *f_0_* of 5.28 GHz.

In addition, we demonstrate that NFP with Meta-NFS can directly integrate multimaterial electronics onto plants (video S5) to create plant bionics that enable in situ monitoring of individual plant environment, growth, and physiological status in real-time (*56*–*58*). Specifically, we printed a wireless capacitive humidity sensor on a leaf (Fig. 6, E and F), which could be used to measure ambient humidity and plant transpiration due to its direct contact with the plant (*59*). At the sensor’s *f_0_* of 3.35 GHz (at 50% relative humidity, RH), the sensor achieves a Q-factor of 11.7 and an average sensitivity of 0.74 MHz/% RH between 50 and 80% RH (root mean squared error: 0.95). Meta-NFS enables the creation of a sensor with a <1 mm^2^ footprint due to its ability to create a freestanding microantenna (video S6) and interface with the temperature-sensitive leaf without the need for an additional print substrate (see fig. S15 for the characterization of adhesion of the graphene trace on the leaf, and see fig. S16 for the cross-sectional scanning electron microscope (SEM) image of printed graphene trace on a leaf). The humidity sensor shows comparable sensitivity with wireless humidity sensors in the literature that operate within a suitable frequency range (fig. S17). In contrast to traditional plant wearable electronics, where the electronics are transferred to the plant postfabrication (*58*–*62*), NFP’s direct printing approach allows for the optimization of sensor geometry and miniaturization of sensor footprint that can reduce potential growth inhibition (e.g., due to limited gas exchange). Meta-NFS’s compatibility with a broad range of functional nanomaterials, including metal and carbon-based materials (*63*), allows us to incorporate inks tailored to desirable attributes. For instance, we integrated graphene with NFP to create a capacitive humidity sensor, leveraging graphene’s high surface area, electron mobility (*64*), and biodegradability (*65*).

## DISCUSSION

In summary, by focusing microwaves to submillimeter resolution with a Meta-NFS, we introduce a 3D printing strategy that enables the creation of spatially freeform, nanomaterial-based electronics—even on temperature-sensitive substrates or within optically opaque materials. Meta-NFS provides both the spatial resolution and power density necessary for electronic 3D printing, allowing rapid and volumetric annealing of functional nanomaterials and polymers that can selectively program a broad range of functional properties. Ultimately, this highly versatile approach expands the capabilities of electronics 3D printing, enabling classes of functional devices that were previously challenging to realize with conventional processes.

To account for variations in substrate and ink dielectric properties that may shift the probe’s resonant frequency, we perform calibration under the prescribed printing conditions to ensure consistent energy coupling and heating efficiency. Moreover, to broaden the approach to inks with low dielectric loss, susceptor materials can be added to increase their absorption of microwave energy. Together, these strategies can further extend the versatility of Meta-NFS, providing a rapid, selective, and volumetric annealing approach that complements existing additive manufacturing approaches.

## MATERIALS AND METHODS

### Materials

Silver nanoparticle ink was synthesized with the protocol inspired by (*8*). Briefly, 1.5 ml of a 50% (w/v) solution of 5-kDa poly(acrylic) acid in water (Polysciences Inc.), 0.84 ml of a 25% solution of 50 kDa poly(acrylic) acid in water (Polysciences Inc.), and 50 ml of distilled water were combined in a 250-ml Erlenmeyer flask. After adding 40 g of diethanolamine (MilliporeSigma), the solution was stirred for 2 hours at 300 rpm using a 1-inch (2.54-cm) stir bar. A silver nitrate solution (20 g of silver nitrate in 20 ml of distilled water) was then added to the poly(acrylic) acid solution while stirring. The solution was covered and stirred for 24 hours, during which time the silver nanoparticles were formed. The nanoparticles were ripened by placing the flask in a 60°C ultrasonic bath for 2 hours. After transferring the nanoparticles to a 500-ml beaker and cooling to room temperature, the nanoparticles were precipitated by titrating 300 ml of ethanol (MilliporeSigma), a poor solvent for the poly(acrylic) acid, at 30 ml/min while stirring. The solution was allowed to settle for 15 min before decanting the supernatant and transferring the silver nanoparticles to a 50-ml centrifuge tube. The nanoparticles were centrifuged at 13,000*g* for 20 min. The remaining supernatant was discarded, and the nanoparticles were mixed with 5 wt % humectant solution (30 wt % ethylene glycol in distilled water) in a centrifugal mixer (Thinky Corp.) for 1 min at 2000 rpm. The ink was placed in a 3-ml syringe barrel (Nordson EFD) and centrifuged at 4000*g* for 10 min to remove air pockets. The syringe barrel was connected to a pulled glass pipette with a 10- to 30-μm inner diameter or a stainless steel dispense tip with a 108-μm inner diameter for printing.

Silicone ink for core-shell NFP was prepared with Dragon Skin (Smooth-On, Inc) with fumed silica. Printing was performed after mixing using a custom core-shell nozzle.

Thermally curing epoxy ink was prepared with two-component heat-cure epoxy (Epoxy Technology Inc.) with 3.5 wt % multiwalled CNTs (Cheap Tubes) and 2 wt % fumed silica (Smooth-On Inc). The components were mixed in a centrifugal mixer (Thinky Corp.) immediately before printing.

Graphene ink was prepared by decreasing the solvent concentration in a gravure printable graphene dispersion (MilliporeSigma). First, 200 mg of graphene dispersion was filtered through a 5-μm syringe filter and placed in a glass petri dish. The petri dish was then placed in a furnace at 150°C (3 min), removed, and the dispersion mixed, cooled (1 min), and mixed again. This process was repeated until the desired rheology was obtained.

Polymeric substrates, including PMMA, UHMW-PE, and silicone (McMaster-Carr), were cleaned with 2-propanol and deionized water before drying with ultrahigh purity nitrogen. Leaf (*Rosa canina*) was rinsed using deionized water and dried using ultrahigh purity nitrogen. Bovine femur bone was obtained from Animal Technologies Inc. Titanium oxide ink was prepared with titania paste (MilliporeSigma), and titanium (IV) oxide nanopowder (MilliporeSigma) was mixed in the weight ratio of 1:2 and ground using a mortar and pestle.

### Core-shell printing assembly

The core-shell outer nozzle was designed using 3D computer-aided design software (SolidWorks) and fabricated with a stereolithography 3D printer (Form 3, Formlabs Co.) using clear resin and adaptive layer height. Core and shell materials were inserted into a 3D-printed (UltiMaker 3) mounting assembly.

### Meta-NFS design and fabrication

The Meta-NFS incorporates a split-ring resonator to enable efficient coupling of microwave-frequency power, while a carefully engineered tapered transmission line further concentrates the coupled energy. As a result, the electrical field generated at the tip of the Meta-NFS can be calculated as follows (*35*)

$$E_{\mathrm{tip}}=4\sqrt{\frac{P_{\text{in}}Z_{0}Q}{\pi d^{2}}}$$

where $P_{\text{in}}$ represents the power fed into the structure, $Z_{0}$ is the characteristic impedance of the microstrip line, Q denotes the Q-factor of the resonator, and d refers to the gap of the tip. The structure was designed and simulated in CST Microwave Studio using a finite-difference time-domain method to evaluate and optimize its generated field strength at the tip. The Meta-NFS was fabricated by micromachining tungsten and then electroplating copper onto the microstrip feedline. The operating frequency of the fabricated Meta-NFS was verified with a Vector Network Analyzer (Keysight).

### Near-field microwave 3D printing

The Meta-NFS and print materials (e.g., core-shell assembly) were mounted to a four-axis micropositioning gantry (Aerotech Inc.). Ink extrusion was controlled using Ultimus V pressure regulators (Nordson EFD). The printing process was executed using a custom Python script to synchronously control the micropositioning gantry, signal generator, pressure regulators, and amplifier power supply. Printing was performed at ambient room temperature (22° to 25°C) and humidity (20 to 30% RH).

### Measurements

To obtain resistivity measurements, linear traces were first printed and annealed on a low-loss substrate. Four-point probe measurements were performed using a source-measure unit (Keithley) attached to a probe station (Micromanipulator Co.). Liquid gallium-indium (MilliporeSigma) was applied to the probing tips to ensure contact with the printed traces. The cross-sectional area was measured using an OLS5100 laser-scanning microscope (Olympus).

Wireless resonant frequency measurements were obtained with the scattering parameter S_11_ of a planar, single-loop inductor, and coaxial cable (N-SMA, 50 ohm, Mini-Circuits) using a handheld radio frequency analyzer (N9912A FieldFox, Keysight). The single-loop inductor was positioned 0.3 to 2 mm from the wireless sensor, and frequencies between 1 and 6 GHz were sampled.

Tensile tests of printed traces and coils were performed using a four-axis micropositioning gantry (Aerotech Inc.) equipped with a 100-g load cell (108AA, Anyload). The two ends of each sample were mounted on the gantry, which controlled the displacement, while the load cell simultaneously recorded the applied force. The displacement and strain data were obtained through image analysis.

### Imaging and videography

Optical images and supporting videos of NFP were acquired with digital microscopes mounted to the four-axis micropositioning gantry. Photographs of printed structures were taken with a digital SLR camera (Canon EOS R6, Canon USA Inc.). The fingerprint shown in Fig. 6E was distorted for privacy using Photoshop (Adobe Systems Inc.). Temperature data were obtained with an IR camera (FLIR AX5 or FLIR T420 with macro lens, Teledyne FLIR).

Cross-sectional SEM images of printed silver nanomaterials were obtained by first scoring the bottom of a PMMA substrate, printing silver nanomaterials above the scored region, and then fracturing the PMMA along this region. The samples were sputter coated with 20 nm of gold-palladium using an EM ACE600 Sputter Coater (Leica) before imaging with the Quanta 600F (FEI) using a 5 to 20 kV electron beam.

### Simulation

We conducted electromagnetic simulations using CST Studio Suite (Dassault Systems). The Meta-NFS probe was modeled as tungsten, while the substrate was Rogers 4350B with the dielectric constant $\epsilon_{r}=3.66$ and loss tangent tanδ=0.0037 . Field distributions were computed through the finite-difference time-domain method, with the probe excited via a waveguide port at varying power inputs. The probe’s excitation efficiency was evaluated through extracted reflection coefficients.

The heat transfer simulations were performed using COMSOL Multiphysics 6.2 with the geometric setup of the Meta-NFS, polyethylene substrate, and ink material identical to that used in the experiments. The simulation used Heat Transfer in Fluids and Solids and Electromagnetic Wave modules. The bottom surface of the substrate was thermally insulated, while all exposed surfaces experienced convective heat loss to the ambient environment, and the Meta-NFS held stationary. We used lumped-port boundary conditions on the dielectric material region between conductive tungsten and copper ground to apply power inputs, and the airbox surfaces were considered scattering boundary conditions.

The silver ink, a mixture of silver nanoparticles and dielectric solvent, was considered an effective medium with a complex permittivity of $\epsilon_{r}=53.5-j15.6$ and modeled as a cylindrical shape with a diameter of 0.03 mm and a length of 2 mm. Ink material properties used for simulations included an electrical conductivity of 0.0038 S/m, a thermal conductivity of 1 W/(m·K), and a density of 10490 kg/m^3^. For the substrate, a thermal conductivity of 0.38 W/(m· K) was applied.

## Supplementary Materials

### The PDF file includes:

Supplementary Text Figs. S1 to S17 Table S1 Legends for movies S1 to S7 References

## References

[1] B. Elder, R. Neupane, E. Tokita, U. Ghosh, S. Hales, Y. L. Kong, Nanomaterial patterning in 3D printing. Adv. Mater. 32, 1907142 (2020). 10.1002/adma.201907142 32129917

[2] F. Han, S. Gu, A. Klimas, N. Zhao, Y. Zhao, S.-C. Chen, Three-dimensional nanofabrication via ultrafast laser patterning and kinetically regulated material assembly. Science 378, 1325–1331 (2022). 36548430 10.1126/science.abm8420

[3] S. Pinilla, J. Coelho, K. Li, J. Liu, V. Nicolosi, Two-dimensional material inks. Nat. Rev. Mater. 7, 717–735 (2022).

[4] S.-F. Liu, Z.-W. Hou, L. Lin, F. Li, Y. Zhao, X.-Z. Li, H. Zhang, H.-H. Fang, Z. Li, H.-B. Sun, 3D nanoprinting of semiconductor quantum dots by photoexcitation-induced chemical bonding. Science 377, 1112–1116 (2022). 36048954 10.1126/science.abo5345

[5] Y. L. Kong, I. A. Tamargo, H. Kim, B. N. Johnson, M. K. Gupta, T.-W. Koh, H.-A. Chin, D. A. Steingart, B. P. Rand, M. C. McAlpine, 3D printed quantum dot light-emitting diodes. Nano Lett. 14, 7017–7023 (2014). 25360485 10.1021/nl5033292

[6] J. Bauer, C. Crook, T. Baldacchini, A sinterless, low-temperature route to 3D print nanoscale optical-grade glass. Science 380, 960–966 (2023). 37262172 10.1126/science.abq3037

[7] M. Zeng, Y. Du, Q. Jiang, N. Kempf, C. Wei, M. V. Bimrose, A. N. M. Tanvir, H. Xu, J. Chen, D. J. Kirsch, J. Martin, B. C. Wyatt, T. Hayashi, M. Saeidi-Javash, H. Sakaue, B. Anasori, L. Jin, M. D. McMurtrey, Y. Zhang, High-throughput printing of combinatorial materials from aerosols. Nature 617, 292–298 (2023). 37165239 10.1038/s41586-023-05898-9 PMC10172128

[8] B. Y. Ahn, E. B. Duoss, M. J. Motala, X. Guo, S.-I. Park, Y. Xiong, J. Yoon, R. G. Nuzzo, J. A. Rogers, J. A. Lewis, Omnidirectional printing of flexible, stretchable, and spanning silver microelectrodes. Science 323, 1590–1593 (2009). 19213878 10.1126/science.1168375

[9] J. U. Lind, T. A. Busbee, A. D. Valentine, F. S. Pasqualini, H. Yuan, M. Yadid, S.-J. Park, A. Kotikian, A. P. Nesmith, P. H. Campbell, J. J. Vlassak, J. A. Lewis, K. K. Parker, Instrumented cardiac microphysiological devices via multimaterial three-dimensional printing. Nat. Mater. 16, 303–308 (2017). 27775708 10.1038/nmat4782 PMC5321777

[10] A. D. Valentine, T. A. Busbee, J. W. Boley, J. R. Raney, A. Chortos, A. Kotikian, J. D. Berrigan, M. F. Durstock, J. A. Lewis, Hybrid 3D printing of soft electronics. Adv. Mater. 29, 1703817 (2017). 10.1002/adma.201703817 28875572

[11] S. B. Walker, J. A. Lewis, Reactive silver inks for patterning high-conductivity features at mild temperatures. J. Am. Chem. Soc. 134, 1419–1421 (2012). 22220580 10.1021/ja209267c

[12] M. Grouchko, A. Kamyshny, C. F. Mihailescu, D. F. Anghel, S. Magdassi, Conductive inks with a “built-in” mechanism that enables sintering at room temperature. ACS Nano 5, 3354–3359 (2011). 21438563 10.1021/nn2005848

[13] M. A. Skylar-Scott, S. Gunasekaran, J. A. Lewis, Laser-assisted direct ink writing of planar and 3D metal architectures. Proc. Natl. Acad. Sci. U.S.A. 113, 6137–6142 (2016). 27185932 10.1073/pnas.1525131113 PMC4896727

[14] Y. Zhao, J. Zhu, W. He, Y. Liu, X. Sang, R. Liu, 3D printing of unsupported multi-scale and large-span ceramic via near-infrared assisted direct ink writing. Nat. Commun. 14, 2381 (2023). 37185359 10.1038/s41467-023-38082-8 PMC10130026

[15] M. S. Brown, C. B. Arnold, “Fundamentals of laser-material interaction and application to multiscale surface modification” in *Laser Precision Microfabrication* (Springer-Verlag, 2010), pp. 91–120.

[16] M. Oghbaei, O. Mirzaee, Microwave versus conventional sintering: A review of fundamentals, advantages and applications. J. Alloys Compd. 494, 175–189 (2010).

[17] C. Leonelli, P. Veronesi, L. Denti, A. Gatto, L. Iuliano, Microwave assisted sintering of green metal parts. J. Mater. Process. Tech. 205, 489–496 (2008).

[18] M. Zhang, S. Fang, A. A. Zakhidov, S. B. Lee, A. E. Aliev, C. D. Williams, K. R. Atkinson, R. H. Baughman, Strong, transparent, multifunctional, carbon nanotube sheets. Science 309, 1215–1219 (2005). 16109875 10.1126/science.1115311

[19] C. B. Sweeney, B. A. Lackey, M. J. Pospisil, T. C. Achee, V. K. Hicks, A. G. Moran, B. R. Teipel, M. A. Saed, M. J. Green, Welding of 3D-printed carbon nanotube–polymer composites by locally induced microwave heating. Sci. Adv. 3, e1700262 (2017). 28630927 10.1126/sciadv.1700262 PMC5470831

[20] Z.-H. Cheng, T. Li, L. Hu, X. Ma, F. Liang, D. Zhao, B.-Z. Wang, Selectively powering multiple small-size devices spaced at diffraction limited distance with point-focused electromagnetic waves. IEEE Trans Ind Electron 69, 13696–13705 (2022).

[21] J. S. Ho, A. J. Yeh, E. Neofytou, S. Kim, Y. Tanabe, B. Patlolla, R. E. Beygui, A. S. Y. Poon, Wireless power transfer to deep-tissue microimplants. Proc. Natl. Acad. Sci. U.S.A. 111, 7974–7979 (2014). 24843161 10.1073/pnas.1403002111 PMC4050616

[22] W. Li, Q. Yu, J. H. Qiu, J. Qi, Intelligent wireless power transfer via a 2-bit compact reconfigurable transmissive-metasurface-based router. Nat. Commun. 15, 2807 (2024). 38561373 10.1038/s41467-024-46984-4 PMC10984985

[23] A. Imtiaz, T. M. Wallis, P. Kabos, Near-field scanning microwave microscopy: An emerging research tool for nanoscale metrology. IEEE Microw. Mag. 15, 52–64 (2014).

[24] K. Hall, H. Zhang, C. Furse, Design of an interstitial microwave applicator for 3D printing in the body. IEEE J. Electromagn. RF Microw.Med. Biol. 4, 260–264 (2019).

[25] A. Shelef, E. Jerby, Incremental solidification (toward 3D-printing) of metal powders by transistor-based microwave applicator. Mater. Des. 185, 108234 (2020).

[26] A. Sarmah, S. K. Desai, A. G. Crowley, G. C. Zolton, G. B. Tezel, E. M. Harkin, T. Q. Tran, K. Arole, M. J. Green, Additive manufacturing of nanotube-loaded thermosets via direct ink writing and radio-frequency heating and curing. Carbon 200, 307–316 (2022).

[27] A. Sarmah, S. K. Desai, G. B. Tezel, A. Vashisth, M. M. Mustafa, K. Arole, A. G. Crowley, M. J. Green, Rapid manufacturing via selective radio-frequency heating and curing of thermosetting resins. Adv. Eng. Mater. 24, 2101351 (2022).

[28] M. Kadic, G. W. Milton, M. van Hecke, M. Wegener, 3D metamaterials. Nat. Rev. Phys. 1, 198–210 (2019).

[29] R. A. Shelby, D. R. Smith, S. Schultz, Experimental verification of a negative index of refraction. Science 292, 77–79 (2001). 11292865 10.1126/science.1058847

[30] S. Głowniak, B. Szczęśniak, J. Choma, M. Jaroniec, Advances in microwave synthesis of nanoporous materials. Adv. Mater. 33, 2103477 (2021). 10.1002/adma.202103477 34580939

[31] A. M. Schwenke, S. Hoeppener, U. S. Schubert, Synthesis and modification of carbon nanomaterials utilizing microwave heating. Adv. Mater. 27, 4113–4141 (2015). 26087742 10.1002/adma.201500472

[32] O. Balci, E. O. Polat, N. Kakenov, C. Kocabas, Graphene-enabled electrically switchable radar-absorbing surfaces. Nat. Commun. 6, 6628 (2015). 25791719 10.1038/ncomms7628

[33] C. A. Balanis, *Antenna Theory: Analysis and Design* (John Wiley & Sons, 2016).

[34] M. Schnell, P. Alonso-González, L. Arzubiaga, F. Casanova, L. E. Hueso, A. Chuvilin, R. Hillenbrand, Nanofocusing of mid-infrared energy with tapered transmission lines. Nat. Photonics 5, 283–287 (2011).

[35] J. Hopwood, F. Iza, S. Coy, D. B. Fenner, A microfabricated atmospheric-pressure microplasma source operating in air. J. Phys. D Appl. Phys. 38, 1698 (2005).

[36] R. Roy, D. Agrawal, J. Cheng, S. Gedevanishvili, Full sintering of powdered-metal bodies in a microwave field. Nature 401, 304–304 (1999).

[37] J. Cheng, R. Roy, D. Agrawal, Radically different effects on materials by separated microwave electric and magnetic fields. Mater. Res. Innov. 5, 170–177 (2002).

[38] Y. Zhang, D. K. Agrawal, J. Cheng, T. Slawecki, Microwave power absorption mechanism of metallic powders. IEEE Trans. Microw. Theory Tech. 66, 2107–2115 (2018).

[39] D. Palessonga, M. E. Gibari, S. Ginestar, H. Terrisse, B. Guiffard, A. Kassiba, H. W. Li, Bandwidth improvement of microwave photonic components based on electro-optic polymers loaded with TiO2 nanoparticles. Appl. Phys. A 123, 542 (2017).

[40] P. P. Urone, R. Hinrichs, “Ch. 19. Electric potential and electric field,” in *College Physics 2e* (OpenStax, 2022), pp. 817–850.

[41] M. Mehdizadeh, *Microwave/RF Applicators and Probes (Second Edition)* (William Andrew, 2015).

[42] M. A. Lieberman, A. J. Lichtenberg, *Principles of Plasma Discharges and Materials Processing* (John Wiley & Sons, ed. 2, 2005).

[43] Y. Sui, C. A. Zorman, R. M. Sankaran, Plasmas for additive manufacturing. Plasma Processes Polym. 17, 2000009 (2020).

[44] D. T. Nguyen, C. Meyers, T. D. Yee, N. A. Dudukovic, J. F. Destino, C. Zhu, E. B. Duoss, T. F. Baumann, T. Suratwala, J. E. Smay, R. Dylla-Spears, 3D-printed transparent glass. Adv. Mater. 29, 1701181 (2017). 10.1002/adma.201701181 28452163

[45] R. M. Cywar, N. A. Rorrer, C. B. Hoyt, G. T. Beckham, E. Y.-X. Chen, Bio-based polymers with performance-advantaged properties. Nat. Rev. Mater. 7, 83–103 (2022).

[46] M. Li, A. Pal, A. Aghakhani, A. Pena-Francesch, M. Sitti, Soft actuators for real-world applications. Nat. Rev. Mater. 7, 235–249 (2022). 35474944 10.1038/s41578-021-00389-7 PMC7612659

[47] P. Wang, M. Hu, H. Wang, Z. Chen, Y. Feng, J. Wang, W. Ling, Y. Huang, The evolution of flexible electronics: From nature, beyond nature, and to nature. Adv. Sci. 7, 2001116 (2020). 10.1002/advs.202001116 PMC7578875 33101851

[48] N. A. Patil, J. Njuguna, B. Kandasubramanian, UHMWPE for biomedical applications: Performance and functionalization. Eur. Polym. J. 125, 109529 (2020).

[49] Z. Hui, L. Zhang, G. Ren, G. Sun, H. Yu, W. Huang, Green flexible electronics: Natural materials, fabrication, and applications. Adv. Mater. 35, 2211202 (2023). 10.1002/adma.202211202 36763956

[50] I. D. Robertson, M. Yourdkhani, P. J. Centellas, J. E. Aw, D. G. Ivanoff, E. Goli, E. M. Lloyd, L. M. Dean, N. R. Sottos, P. H. Geubelle, J. S. Moore, S. R. White, Rapid energy-efficient manufacturing of polymers and composites via frontal polymerization. Nature 557, 223–227 (2018). 29743687 10.1038/s41586-018-0054-x

[51] M. Chen, Z. Zhou, S. Hu, N. Huang, H. Lee, Y. Liu, J. Yang, X. Huan, Z. Xu, S. Cao, X. Cheng, T. Wang, S. F. Yu, B. P. Chan, J. Tang, S. Feng, J. T. Kim, 3D printing of arbitrary perovskite nanowire heterostructures. Adv. Funct. Mater. 33, 2212146 (2023).

[52] Y. Park, I. Yun, W. G. Chung, W. Park, D. H. Lee, J. Park, High-resolution 3D printing for electronics. Adv. Sci. 9, 2104623 (2022). 10.1002/advs.202104623 PMC8922115 35038249

[53] J. J. Adams, S. C. Slimmer, J. A. Lewis, J. T. Bernhard, 3D-printed spherical dipole antenna integrated on small RF node. Electron. Lett. 51, 661–662 (2015).

[54] C. Pfeiffer, X. Xu, S. R. Forrest, A. Grbic, Direct transfer patterning of electrically small antennas onto three-dimensionally contoured substrates. Adv. Mater. 24, 1166–1170 (2012). 22290732 10.1002/adma.201104290

[55] P. K. Sharma, N. Gupta, P. I. Dankov, Characterization of polydimethylsiloxane (PDMS) as a wearable antenna substrate using resonance and planar structure methods. AEU - Int. J. Electron. Commun. 127, 153455 (2020).

[56] R. Rayhana, G. G. Xiao, Z. Liu, Printed sensor technologies for monitoring applications in smart farming: A review. IEEE Trans. Instrum. Meas. 70, 1–19 (2021).33776080

[57] T. T. S. Lew, V. B. Koman, P. Gordiichuk, M. Park, M. S. Strano, The emergence of plant nanobionics and living plants as technology. Adv. Mater. Technol. 5, 1900657 (2020).

[58] J. P. Giraldo, H. Wu, G. M. Newkirk, S. Kruss, Nanobiotechnology approaches for engineering smart plant sensors. Nat. Nanotechnol. 14, 541–553 (2019). 31168083 10.1038/s41565-019-0470-6

[59] Y. Lu, K. Xu, L. Zhang, M. Deguchi, H. Shishido, T. Arie, R. Pan, A. Hayashi, L. Shen, S. Akita, K. Takei, Multimodal plant healthcare flexible sensor system. ACS Nano 14, 10966–10975 (2020). 32806070 10.1021/acsnano.0c03757

[60] J. M. Nassar, S. M. Khan, D. R. Villalva, M. M. Nour, A. S. Almuslem, M. M. Hussain, Compliant plant wearables for localized microclimate and plant growth monitoring. NPJ Flexible Electron. 2, 24 (2018).

[61] S. Yin, H. Ibrahim, P. S. Schnable, M. J. Castellano, L. Dong, A field-deployable, wearable leaf sensor for continuous monitoring of vapor-pressure deficit. Adv. Mater. Technol. 6, 2001246 (2021).

[62] S. Wang, Y. Diao, Printed electronics for cultivating plants in space. Nat. Rev. Mater. 9, 762–763 (2024).

[63] E. Vázquez, M. Prato, Carbon nanotubes and microwaves: Interactions, responses, and applications. ACS Nano 3, 3819–3824 (2009). 20025299 10.1021/nn901604j

[64] J. P. Mensing, T. Lomas, A. Tuantranont, 2D and 3D printing for graphene based supercapacitors and batteries: A review. Sustain. Mater. Technol. 25, e00190 (2020).

[65] M. Chen, X. Qin, G. Zeng, Biodegradation of carbon nanotubes, graphene, and their derivatives. Trends Biotechnol. 35, 836–846 (2017). 28063621 10.1016/j.tibtech.2016.12.001

[66] E. Jerby, Y. Meir, A. Salzberg, E. Aharoni, A. Levy, J. P. Torralba, B. Cavallini, Incremental metal-powder solidification by localized microwave-heating and its potential for additive manufacturing. Addit. Manuf. 6, 53–66 (2015).

[67] X. Qi, J. Xu, Q. Hu, Y. Deng, R. Xie, Y. Jiang, W. Zhong, Y. Du, Metal-free carbon nanotubes: Synthesis, and enhanced intrinsic microwave absorption properties. Sci. Rep. 6, 28310 (2016). 27324290 10.1038/srep28310 PMC4915013

[68] J. Liu, Z. Jia, W. Zhou, X. Liu, C. Zhang, B. Xu, G. Wu, Self-assembled MoS_2_/magnetic ferrite CuFe_2_O_4_ nanocomposite for high-efficiency microwave absorption. Chem. Eng. J. 429, 132253 (2022).

[69] M. Green, P. Xiang, Z. Liu, J. Murowchick, X. Tan, F. Huang, X. Chen, Microwave absorption of aluminum/hydrogen treated titanium dioxide nanoparticles. J. Mater. 5, 133–146 (2019).

[70] H. Bayrakdar, Complex permittivity, complex permeability and microwave absorption properties of ferrite–paraffin polymer composites. J. Magn. Magn. Mater. 323, 1882–1885 (2011).

[71] Z. Dong, M. Vuckovac, W. Cui, Q. Zhou, R. H. A. Ras, P. A. Levkin, 3D printing of superhydrophobic objects with bulk nanostructure. Adv. Mater. 33, 2106068 (2021). 34580937 10.1002/adma.202106068 PMC11468021

[72] H. Wang, Q. Ruan, H. Wang, S. D. Rezaei, K. T. P. Lim, H. Liu, W. Zhang, J. Trisno, J. Y. E. Chan, J. K. W. Yang, Full color and grayscale painting with 3D printed low-index nanopillars. Nano Lett. 21, 4721–4729 (2021). 34019769 10.1021/acs.nanolett.1c00979

[73] M. Ali, F. Alam, N. Vahdati, H. Butt, 3D-printed holographic fresnel lenses. Adv. Eng. Mater. 24, 2101641 (2022).

[74] Y. Mu, K. Sun, Y. Jia, N. Zhang, S. Wu, Y. Jia, G. Wang, 3D-printed strong and ductile high-entropy alloys with orientation arranged nanostructure complex. J. Alloys Compd. 968, 171824 (2023).

[75] B. Weidinger, G. Yang, N. von Coelln, H. Nirschl, I. Wacker, P. Tegeder, R. R. Schröder, E. Blasco, 3D printing hierarchically nano-ordered structures. Adv. Sci. 10, 2302756 (2023). 10.1002/advs.202302756 PMC10558687 37532671

[76] A. Albrecht, A. Rivadeneyra, A. Abdellah, P. Lugli, J. F. Salmerón, Inkjet printing and photonic sintering of silver and copper oxide nanoparticles for ultra-low-cost conductive patterns. J. Mater. Chem. C 4, 3546–3554 (2016).

[77] Y.-T. Kwon, Y.-S. Kim, Y. Lee, S. Kwon, M. Lim, Y. Song, Y.-H. Choa, W.-H. Yeo, Ultrahigh conductivity and superior interfacial adhesion of a nanostructured, photonic-sintered copper membrane for printed flexible hybrid electronics. ACS Appl. Mater. Interfaces 10, 44071–44079 (2018). 30452228 10.1021/acsami.8b17164

[78] S. Majee, W. Zhao, A. Sugunan, T. Gillgren, J. A. Larsson, R. Brooke, N. Nordgren, Z. Zhang, S. Zhang, D. Nilsson, A. Ahniyaz, Highly conductive films by rapid photonic annealing of inkjet printable starch–graphene ink. Adv. Mater. Interfaces 9, 2101884 (2022).

[79] Y. Kim, H. Yuk, R. Zhao, S. A. Chester, X. Zhao, Printing ferromagnetic domains for untethered fast-transforming soft materials. Nature 558, 274–279 (2018). 29899476 10.1038/s41586-018-0185-0

[80] F. Kotz, K. Arnold, W. Bauer, D. Schild, N. Keller, K. Sachsenheimer, T. M. Nargang, C. Richter, D. Helmer, B. E. Rapp, Three-dimensional printing of transparent fused silica glass. Nature 544, 337–339 (2017). 28425999 10.1038/nature22061

[81] M. S. Saleh, C. Hu, R. Panat, Three-dimensional microarchitected materials and devices using nanoparticle assembly by pointwise spatial printing. Sci. Adv. 3, e1601986 (2017). 28275733 10.1126/sciadv.1601986 PMC5336350

[82] T. J. K. Buchner, S. Rogler, S. Weirich, Y. Armati, B. G. Cangan, J. Ramos, S. T. Twiddy, D. M. Marini, A. Weber, D. Chen, G. Ellson, J. Jacob, W. Zengerle, D. Katalichenko, C. Keny, W. Matusik, R. K. Katzschmann, Vision-controlled jetting for composite systems and robots. Nature 623, 522–530 (2023). 37968527 10.1038/s41586-023-06684-3 PMC10651485

[83] M. A. Skylar-Scott, J. Mueller, C. W. Visser, J. A. Lewis, Voxelated soft matter via multimaterial multinozzle 3D printing. Nature 575, 330–335 (2019). 31723289 10.1038/s41586-019-1736-8

[84] W. Jung, Y.-H. Jung, P. V. Pikhitsa, J. Feng, Y. Yang, M. Kim, H.-Y. Tsai, T. Tanaka, J. Shin, K.-Y. Kim, H. Choi, J. Rho, M. Choi, Three-dimensional nanoprinting via charged aerosol jets. Nature 592, 54–59 (2021). 33790446 10.1038/s41586-021-03353-1

[85] S. Ling, X. Tian, Q. Zeng, Z. Qin, S. A. Kurt, Y. J. Tan, J. Y. H. Fuh, Z. Liu, M. D. Dickey, J. S. Ho, B. C. K. Tee, Tension-driven three-dimensional printing of free-standing field’s metal structures. Nat. Electron. 7, 671–683 (2024).

[86] M.-Z. Xie, L.-F. Wang, L. Dong, W.-J. Deng, Q.-A. Huang, Low cost paper-based LC wireless humidity sensors and distance-insensitive readout system. IEEE Sensors J. 19, 4717–4725 (2019).

[87] Q.-Y. Ren, L.-F. Wang, J.-Q. Huang, C. Zhang, Q.-A. Huang, Simultaneous remote sensing of temperature and humidity by LC-type passive wireless sensors. J. Microelectromech. Syst. 24, 1117–1123 (2015).

[88] M. Borgese, F. A. Dicandia, F. Costa, S. Genovesi, G. Manara, An inkjet printed chipless RFID sensor for wireless humidity monitoring. IEEE Sensors J. 17, 4699–4707 (2017).

[89] X. Lin, B.-C. Seet, F. Joseph, Wearable humidity sensing antenna for BAN applications over 5G networks. *2018 IEEE 19th Wirel. Microw. Technol. Conf. (WAMICON)* (2018); pp. 1–4.

[90] W. Lv, Y. Zhang, H. Luo, Q. Xu, W. Quan, J. Yang, M. Zeng, N. Hu, Z. Yang, Wide remote-range and accurate wireless LC temperature–humidity sensor enabled by efficient mutual interference mitigation. ACS Sens. 8, 4531–4541 (2023). 38006356 10.1021/acssensors.3c01200

[91] Y. Li, Z. Wei, J. Huang, An LC-type flexible wireless humidity sensor with electrospun isolation layer. 2021 IEEE Sens. 00, 1–4 (2021).

[92] Y.-B. Xue, H.-Y. Huang, W.-J. Zhu, B.-J. Chen, Y.-J. Ju, C. Feng, Wireless, passive paper-based LC humidity sensor with RFID frequency of 13.56 MHz. IEEE Sensors J. 24, 15800–15810 (2024).

[93] S. Su, W. Lv, T. Zhang, Q. Tan, W. Zhang, J. Xiong, A MoS_2_ nanoflakes-based LC wireless passive humidity sensor. Sensors 18, 4466 (2018). 30562969 10.3390/s18124466 PMC6308545

[94] W.-J. Deng, L.-F. Wang, L. Dong, Q.-A. Huang, Experimental study of the bending effect on LC wireless humidity sensors fabricated on flexible PET substrates. J. Microelectromech. Syst. 27, 761–763 (2018).

[95] W. Lv, Q. Tan, H. Kou, W. Zhang, J. Xiong, MWCNTs/WS_2_ nanocomposite sensor realized by LC wireless method for humidity monitoring. Sens. Actuators A: Phys. 290, 207–214 (2019).

[96] E. M. Amin, M. S. Bhuiyan, N. C. Karmakar, B. Winther-Jensen, Development of a low cost printable chipless RFID humidity sensor. IEEE Sensors J. 14, 140–149 (2013).

[97] J.-K. Park, T.-G. Kang, B.-H. Kim, H.-J. Lee, H. H. Choi, J.-G. Yook, Real-time humidity sensor based on microwave resonator coupled with PEDOT:PSS conducting polymer film. Sci. Rep. 8, 439 (2018). 29323214 10.1038/s41598-017-18979-3 PMC5764980

[98] T.-K. Nguyen, C.-H. Tseng, A new microwave humidity sensor with near-field self-injection-locked technology. IEEE Sensors J. 21, 21520–21528 (2021).

[99] C. Zhang, L. Guo, L. Wang, J. Huang, Q. Huang, Passive wireless integrated humidity sensor based on dual-layer spiral inductors. Electron. Lett. 50, 1287–1289 (2014).

[100] C. Zhang, L.-F. Wang, J.-Q. Huang, Q.-A. Huang, An LC-type passive wireless humidity sensor system with portable telemetry unit. J. Microelectromech. Syst. 24, 575–581 (2015).

