# Supplementary material for: Three-dimensional printing of nanomaterials-based electronics with a metamaterial-inspired near-field electromagnetic structure
Source: Sci Adv. 2026 Feb 6;12(6):eadz7415. doi: 10.1126/sciadv.adz7415 (PMC12880538; doi:10.1126/sciadv.adz7415)
Supplement: Supplementary file 1 — Supplementary Text Figs. S1 to S17 Table S1 Legends for movies S1 to S7 References [file sciadv.adz7415_sm.pdf]

Science Advances
AAAS

Supplementary Materials for

Three-dimensional printing of nanomaterials-based electronics with a metamaterial-inspired near-field electromagnetic structure

Jian Teng *et al.*

Corresponding author: John S. Ho, johnho@nus.edu.sg; Yong Lin Kong, kong@rice.edu

Sci. Adv. **12**, eadz7415 (2026)
DOI: 10.1126/sciadv.adz7415

The PDF file includes:

Supplementary Text
Figs. S1 to S17
Table S1
Legends for movies S1 to S7
References

Other Supplementary Material for this manuscript includes the following:

Movies S1 to S7

Supplementary Text

NFP heat transfer mechanism

In the microwave heating process, the absorbed microwave power in the material is converted into heat, leading to volumetric annealing of the ink. The microwave power density absorbed in the materials includes contributions from ohmic, dielectric, and magnetic heating, and can be estimated as (*66*) $Q = Q_{ohm} + Q_{diel} + Q_{mag} = 1/2\,\sigma_e|\mathbf{E}|^2 + 1/2\,\omega\epsilon_0\epsilon_r''|\mathbf{E}|^2 + 1/2\,\omega\mu_0\mu_r''|\mathbf{H}|^2$, where σ_e is the electric conductivity, ω is the angular frequency, **E** is the electric field vector, **H** is the magnetic field vector, ϵ_0 is the vacuum permittivity, ϵ_r'' is the imaginary part of the complex permittivity, μ_0 is the vacuum permeability, and μ_r'' is the imaginary part of the complex permeability.

For non-magnetic materials, the complex permittivity, $\epsilon_r = \epsilon_r' - j\epsilon_r''$, is a key parameter that describes the level of heat generation. Here, the real part ϵ_r', commonly known as the dielectric constant, represents the material's ability to store electric energy, whereas the imaginary part ϵ_r'' is called the dielectric loss factor, which represents the ability of the material to dissipate the stored electric energy as heat. Therefore, microwave heating is particularly effective for materials with high dielectric loss factors, including metal nanoparticles, which contribute to ohmic loss, and the dielectric solvent contributes to dielectric loss.

Temperature distribution in the material can be predicted from the energy equation coupled with the microwave-absorbed power density Q:

$$\rho c_p \frac{\partial T}{\partial t} + \rho c_p \mathrm{u} \cdot \nabla = k\nabla^2 T + Q - Q_{conv},$$

where ρ is the density, c_p is the heat capacity, T is the temperature, t is the time, u is the printing speed in the y direction, k is the thermal conductivity, and Q_{conv} is the convective heat loss. We simulated heat transfer in a silver ink trace of 30 μm diameter on a polyethylene substrate at a print speed of 0.2 mm/s. Here, the magnetic heating was neglected, and the silver ink (mixture of silver nanoparticle and dielectric solvent) was treated as an effective medium. The overall complex permittivity of the silver ink was measured as $\epsilon_r = 53.5 - j15.6$ using a network analyzer.

The energy absorption and heat transfer also depend on the electrical and thermal conductivities of the ink trace. Annealed and unannealed regions of the ink trace experience different conductivity (fig. S4A). Within the annealed region, electric field absorption is suppressed due to its high electrical conductivity. Additionally, heat diffuses further along the annealed region as the thermal conductivity increases from 1 to 50 W/Km, making selective heating more challenging (fig. S4B-C).

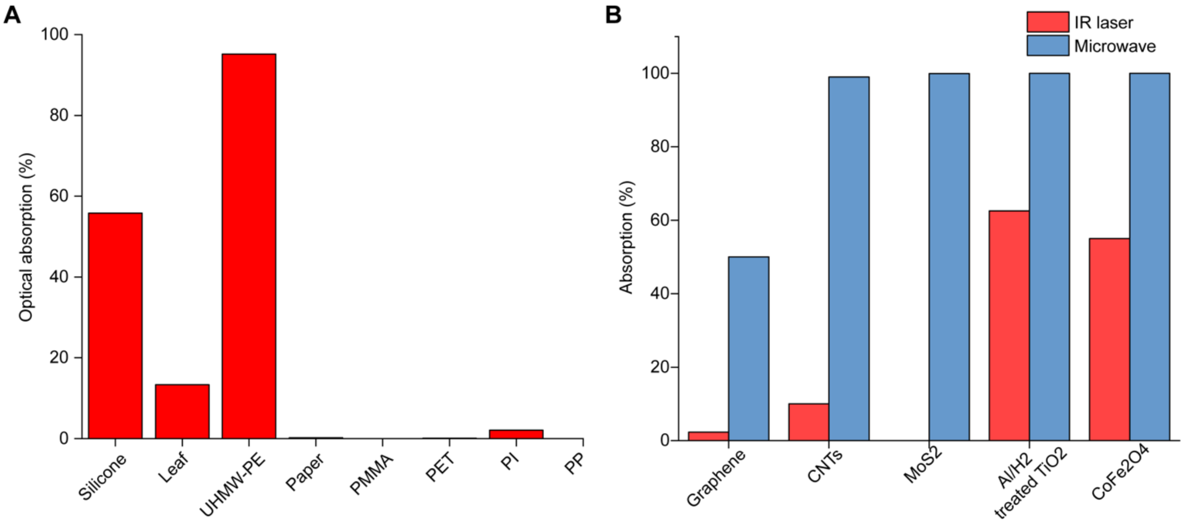

Fig. S1. Optical and microwave absorption of materials. (**A**) Measured optical absorption of select temperature-sensitive substrates at 850 nm. The substrates with high optical absorption used in this work, such as orange silicone, black UHMW-PE, and the leaf, are not well suited for a laser or photonic annealing approach. Substrates shown with low optical absorption are compatible with either a microwave or laser annealing approach. (**B**) Comparison of microwave and laser absorption for various materials from literature (*32*, *67–70*).

Annealing mechanism	Print materials<br>M1. Polymers<br>M2. Metal<br>M3. Ceramics<br>M4. Other				Temperature-sensitive substrates<br>S1. Polymers<br>S2. Paper<br>S3. Plant materials<br>S4. Biological materials				Printing capabilities				Properties and resolution					Reference
	M1	M2	M3	M4	S1	S2	S3	S4	Spanning structures	Freestanding structures	Tunable properties	Multi-material printing	Minimum feature size (μm)	Power (W)	Power (kW/cm^2)	Pulse width (ms)	Print speed (mm/s)	
Microwave	**Y**	**Y**	**Y**	**Y**	**Y**	**Y**	**Y**	**Y**	**Y**	**Y**	**Y**	**Y**	**4**	**14**	**~17.5**	**3-100**	**0.1-0.5**	**This work**
	-	Y	-	-	-	-	-	-	-	-	-	-	2000	<200	-	8000	-	(*25*)
	Y	-	-	-	-	-	-	-	-	-	-	-	800	-	-	continuous	0.067	(*27*)
Laser	-	Y	-	-	Y*	-	-	-	Y	Y	Y	-	<1	-	30	1	0.5-2	(*13*)
	-	-	Y	-	-	-	-	-	Y	Y	-	-	410	-	0.082-0.713	3000	1-41	(*14*)
	Y	Y	Y	Y	-	-	-	-	Y	-	-	-	0.036	-	0.4-0.65	1-80	-	(*2*)
	-	Y	-	Y	-	-	-	-	Y	Y	-	-	0.081	-	4760	$9.80x10^{-11}$	0.002-0.02	(*4*)
	Y	-	-	-	-	-	-	-	-	-	-	-	50	-	$1x10^{-6}$	-	-	(*71*)
	Y	-	-	-	-	-	-	-	-	-	-	-	0.343	0.05	-	$1x10^{-10}$	-	(*72*)
	Y	-	-	-	-	-	-	-	-	-	-	-	5	-	-	-	-	(*73*)
	-	Y	-	-	-	-	-	-	-	-	-	-	50	180	-	-	800	(*74*)
	Y	-	-	-	-	-	-	-	-	-	-	-	0.2	0.0225	-	0.2	2.5	(*75*)
Photonic	-	Y	-	-	Y*	Y	-	-	-	-	-	-	50	-	1.58-3.38	1-2	-	(*76*)
	-	Y	-	-	Y*	-	-	-	-	-	-	-	2000	$2x10^{6}$	-	8-20	-	(*77*)
	-	-	-	Y	Y*	-	-	-	-	-	-	-	60		0.04-3.08	1	-	(*78*)
Oven	Y	Y	-	-	-	-	-	-	-	-	-	-	50	-	-	-	-	(*79*)
	-	-	Y	-	-	-	-	-	-	-	-	-	20	-	-	-	-	(*80*)
	-	Y	-	-	-	-	-	-	Y	Y	-	-	100	-	-	-	-	(*81*)
None	Y	-	-	Y	-	-	-	-	-	-	-	Y	20	-	-	-	0.004	(*82*)
	Y	-	-	Y	-	-	-	-	-	-	-	Y	200	-	-	-	40	(*83*)
	Y	-	-	-	-	-	-	-	Y	Y	-	-	250	-	-	-	1.25	(*50*)
	-	Y	-	-	-	-	-	-	Y	Y	-	Y	0.085	-	-	-	0.0083-0.0667	(*84*)
	-	Y	-	-	Y	Y	-	-	Y	Y	-	-	100	-	-	-	0.01-100	(*85*)

Table. S1. Comparison of NFP with previous 3D printing and annealing approaches.
"-" signifies that no information is available.
Y*: substrates used include polymers with low optical absorption, including polypropylene, polyimide, and polyethylene terephthalate. A comparison of the optical absorption of these substrates and substrates compatible with NFP are shown in fig. S1.

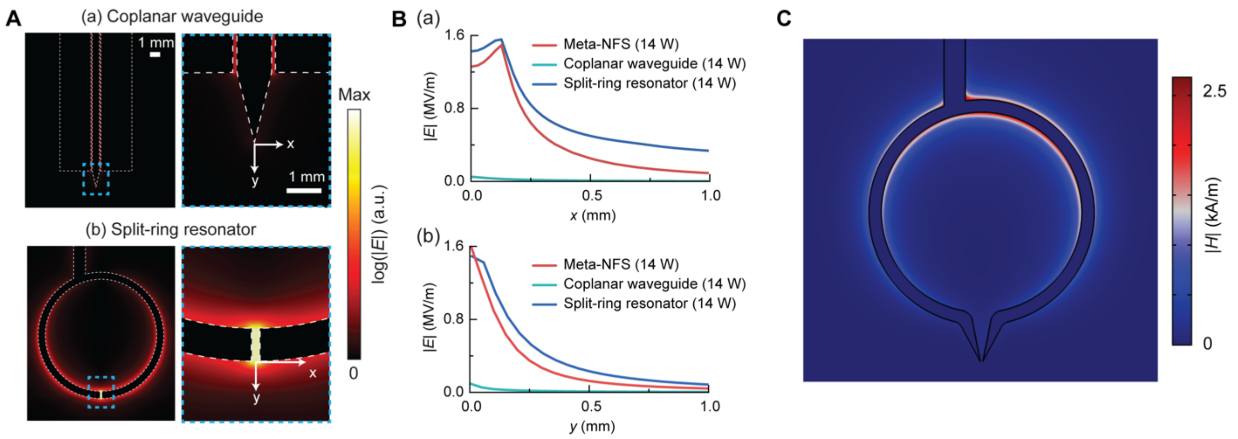

Fig. S2. (A) & (B) Simulated electric field magnitude for the conventional structures: (a) coplanar and (b) split-ring resonator. (C) Simulated magnetic field magnitude of the Meta-NFS.

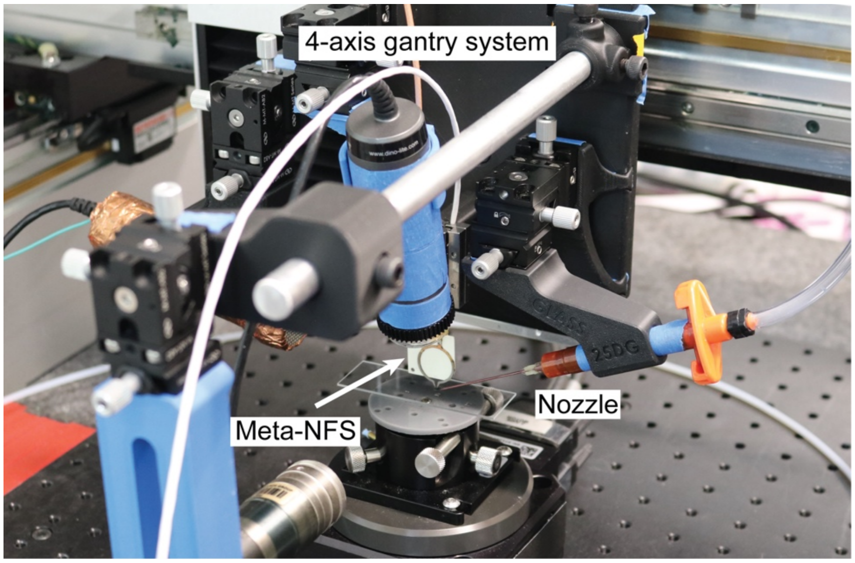

Fig. S3. Photograph of the printing system setup with Meta-NFS.

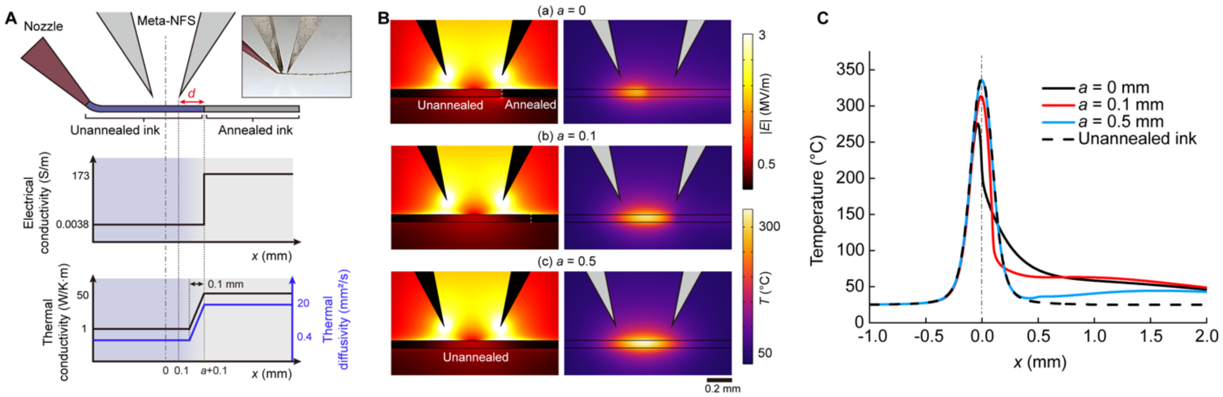

Fig. S4. Simulated electric field magnitude and temperature distribution in unannealed and annealed regions of a trace. (**A**) Electrical conductivity and thermal conductivity profiles used for simulations of unannealed and annealed regions. The electrical conductivities of 0.0038 S/m and 173 S/m were applied to the unannealed and annealed regions, respectively. The thermal conductivity was assumed to increase linearly along the ink trace length (x) from the annealed to the unannealed region. The annealed trace has 50 times higher thermal conductivity and diffusivity than the unannealed trace. (**B**) 2D simulation images showing the electric field magnitude (left) and temperature distribution (right) in the trace for various distances d between the probe tip and annealed ink. Images (a) and (b) illustrate heat transfer through the unannealed and annealed traces. For more electrically conductive material, the electric field is less concentrated, limiting heating in the material. Image (c) represents the scenario where the NFS is positioned above the unannealed region with constant electrical and thermal conductivities. (**C**) Temperature distribution along the x-axis at various probe positions.

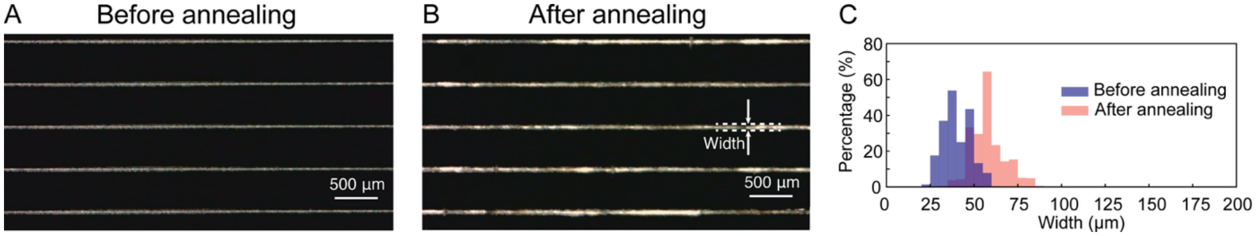

Fig. S5. Printing of silver nanoparticle traces. (**A)** Silver nanoparticle traces before annealing with an average 40 µm width. (**B**) Silver nanoparticle traces after annealing with Meta-NFS with an average 54 µm width. (**C**) Trace width distributions before and after annealing.

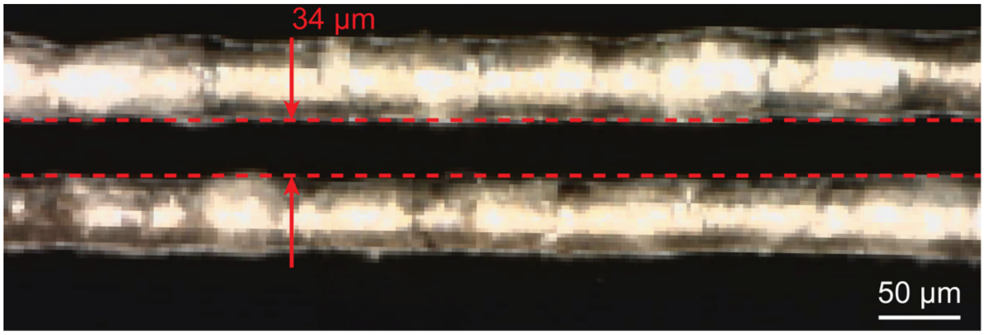

Fig. S6. Minimum spacing that can reliably be achieved with Meta-NFS (200 µm tip spacing) using a 30 µm nozzle.

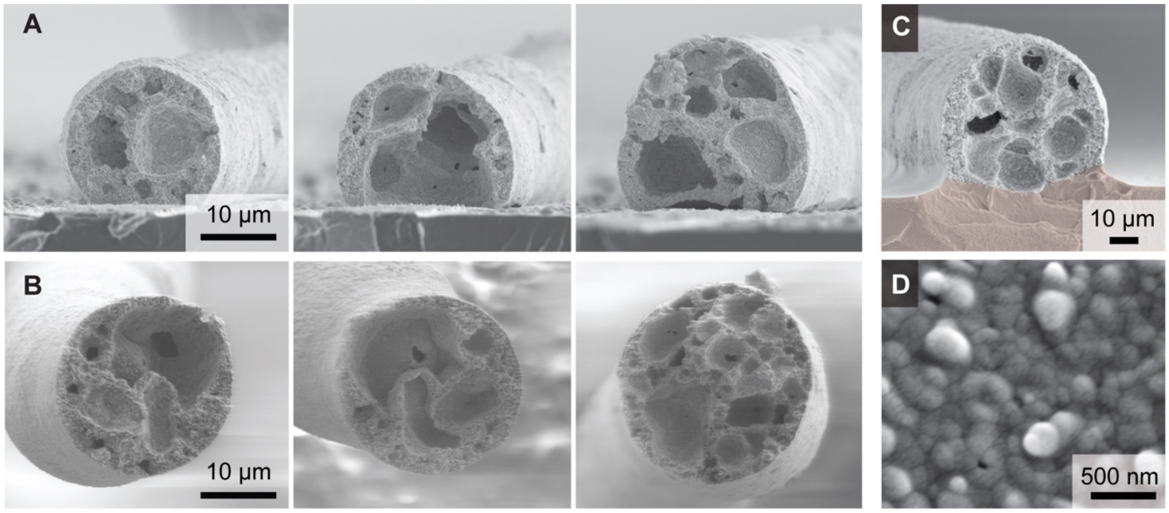

Fig. S7. (**A**) **&** (**B**) **Microstructure of planar and freestanding silver traces.** (**A**) The microstructure of planar silver traces on PMMA (average resistivity: 2.9×10^{-4} ohms·cm) when compared with (**B**) the microstructure of freestanding traces annealed with the same microwave parameters (average resistivity: 9.2×10^{-6} ohms·cm). (**C**) **&** (**D**) **Reduced heat-affected zone in annealed silver traces at higher power with lower pulse width.** (**C**) cross-sectional and (**D**) microscopy images of silver nanomaterial traces annealed to a resistivity of 8.9×10^{-5} ohms·cm using higher power (43.5 W) and lower pulse width (12 ms), compared to the trace annealed at a power of 14 W and a pulse width of 100 ms (resistivity of 5.3×10^{-5} ohms·cm), shown in Fig. 3A, resulting in a reduced heat-affected zone in the polymeric substrate (PMMA, highlighted in red).

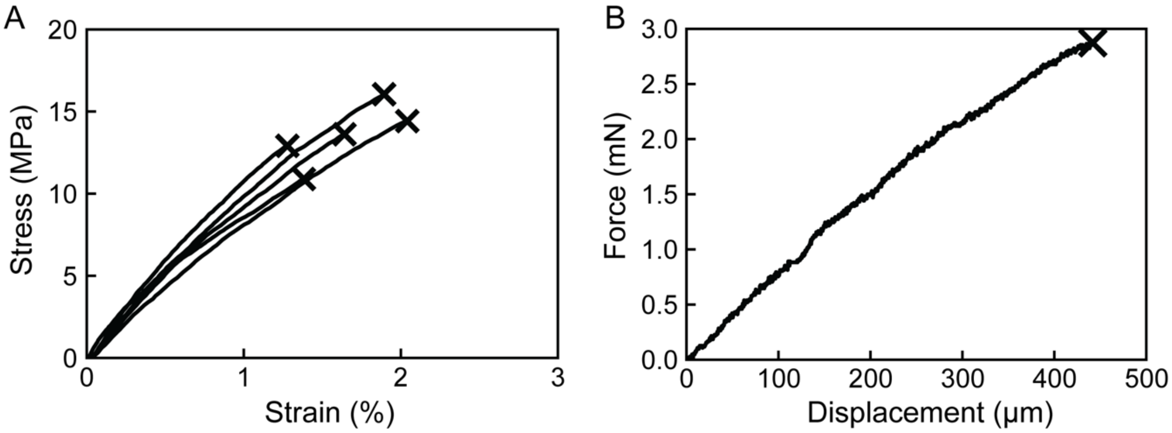

Fig. S8. Tensile test of printed silver trace and silver microspiral. (**A**) Plot of the stress-strain curve for five printed silver traces with a posted sintered diameter of 54 μm. (**B**) Plot of force-displacement curve for the printed silver microspiral with an outer diameter of 1.25 mm. The cross mark indicates the fracture point.

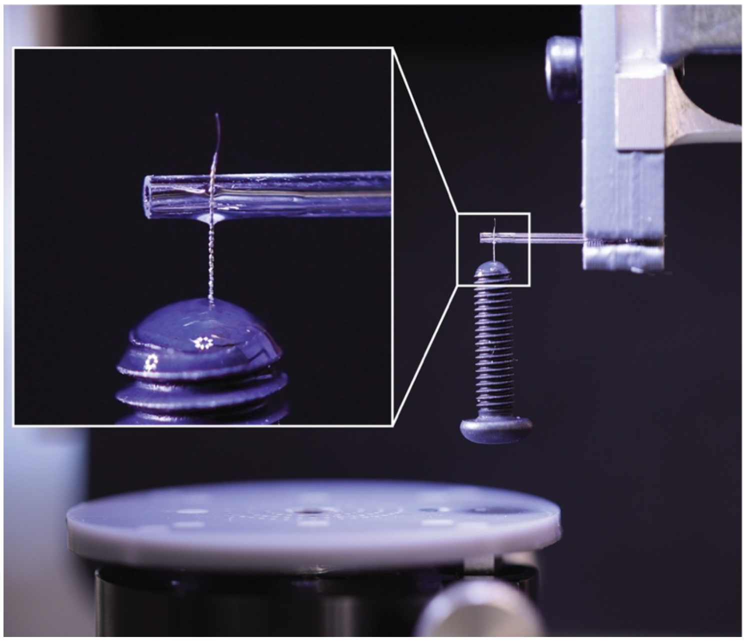

Fig. S9. Demonstration of the mechanical strength of a printed silver trace, in which a silver trace supports a suspended screw (M4, 16 mm, weight: 1.7 g). The inset shows a zoom-in view of the attachment region between the printed trace and the screw. The trace is secured to the screw using UV glue.

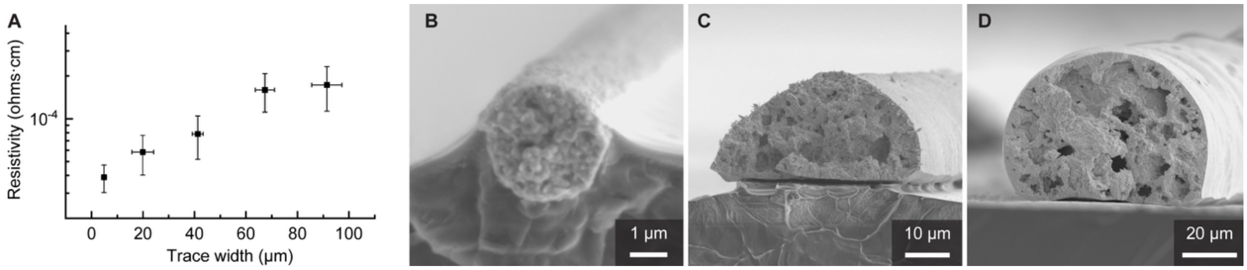

Fig. S10. Effect of silver trace width on electrical resistivity. (**A**) Electrical resistivity of silver traces annealed with the same microwave parameters with respect to printed trace width. The lowest achieved resistivity is 3.88×10^{-5} ohms·cm. (**B**)(**C**)(**D**) SEM images of silver traces with different trace widths. The resistivity was measured for at least three samples for each width. The error bars on the x-axis represent the standard deviation of the trace width, while those on the y-axis represent the standard deviation of the resistivity.

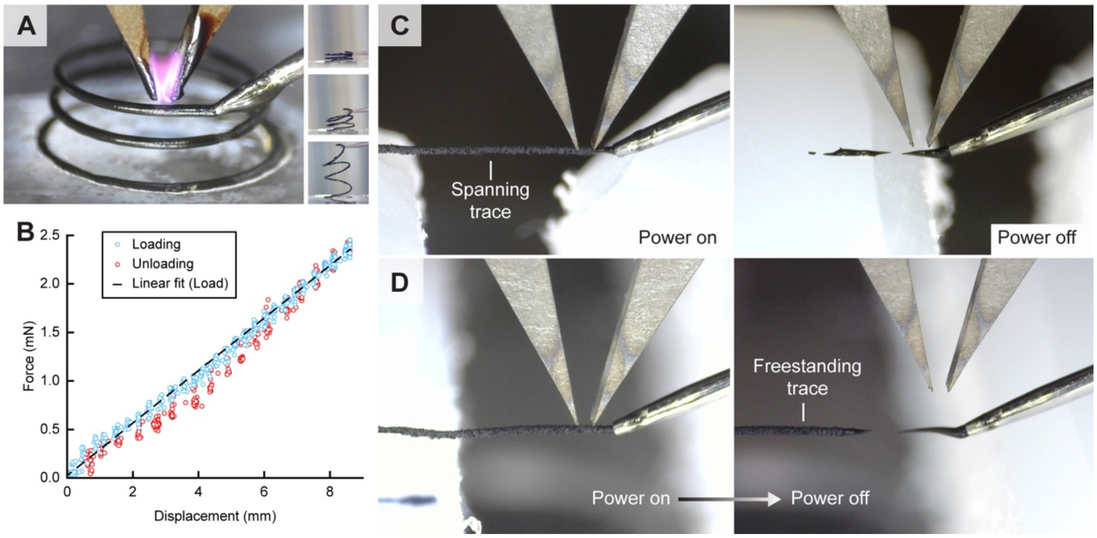

Fig. S11. Selective annealing enables microscale 3D printing of thermoset and graphene inks. (**A**) Photograph demonstrating the selective crosslinking of a thermoset ink with only 3.5 wt% microwave-absorbing nanomaterials to create a freestanding microspring (100 µm trace diameter). The inset shows microspring during elongation. (**B**) The measured force of a printed microspring with respect to displacement, resulting in >250% elastic strain before recovery (spring constant: 0.271 N/m during loading). (**C**) Photographs showing the printing of graphene traces that are capable of spanning a gap when NFP is turned on but incapable of spanning when printed as-is without NFP. The (power on) image is reused from Fig. 3D (graphene). (**D**) Photographs demonstrating in situ control of rheological properties to enable the creation of freestanding architecture. The spanning trace (left) is terminated mid-air (right) to form a freestanding cantilever by modulating the NFP power partway across the gap.

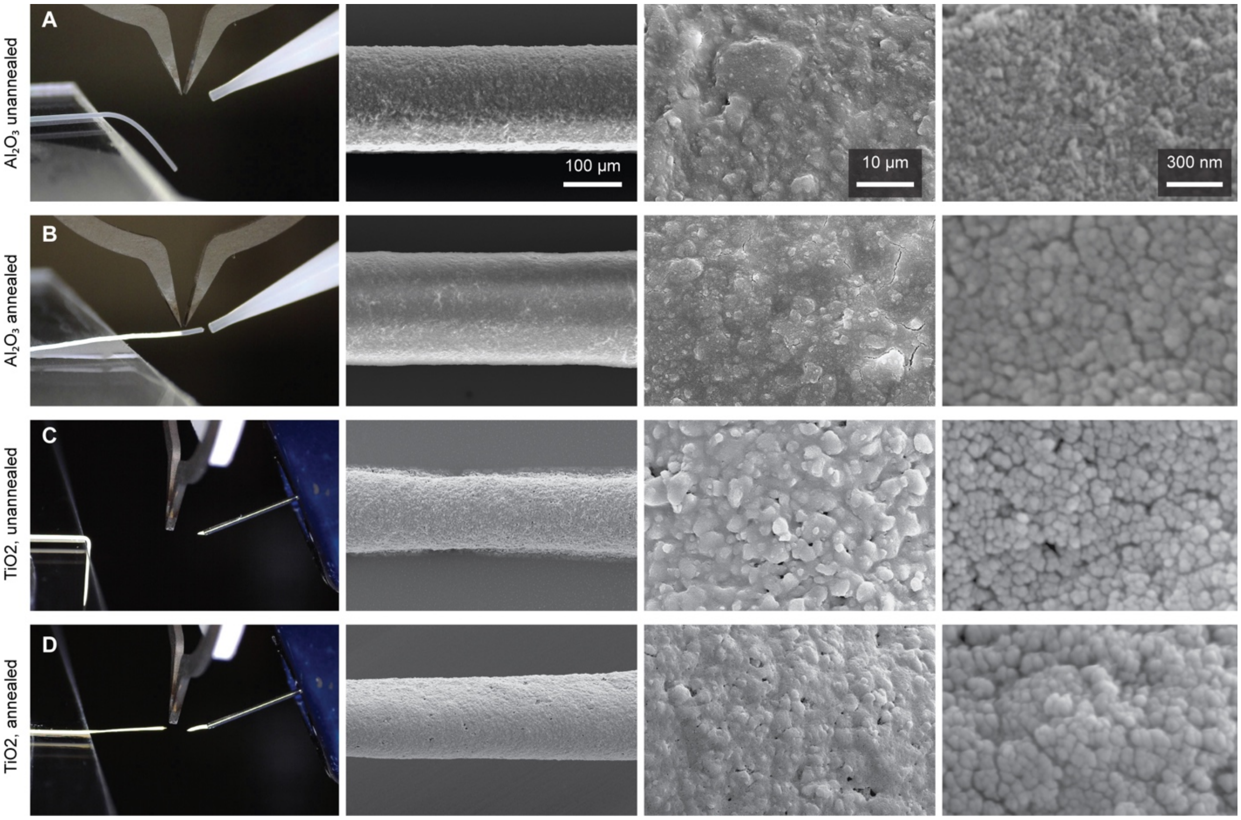

Fig. S12. NFP of ceramic nanomaterial inks. (**A**) (**B**) Photographs showing the printing of aluminum oxide traces that are (**A**) incapable of maintaining a freestanding configuration without NFP but (**B**) capable of freestanding when printed with NFP. SEM images show the microstructure of the traces. The leftmost image in (B) is reused from Fig. 3D (Al_2O_3). (**C**) (**D**) Photographs showing the printing of titanium oxide traces that are (**C**) incapable of maintaining a freestanding configuration without NFP but (**D**) capable of freestanding when printed with NFP. SEM images show the microstructure of the traces. The leftmost image in (D) is reused from Fig. 3D (TiO_2).

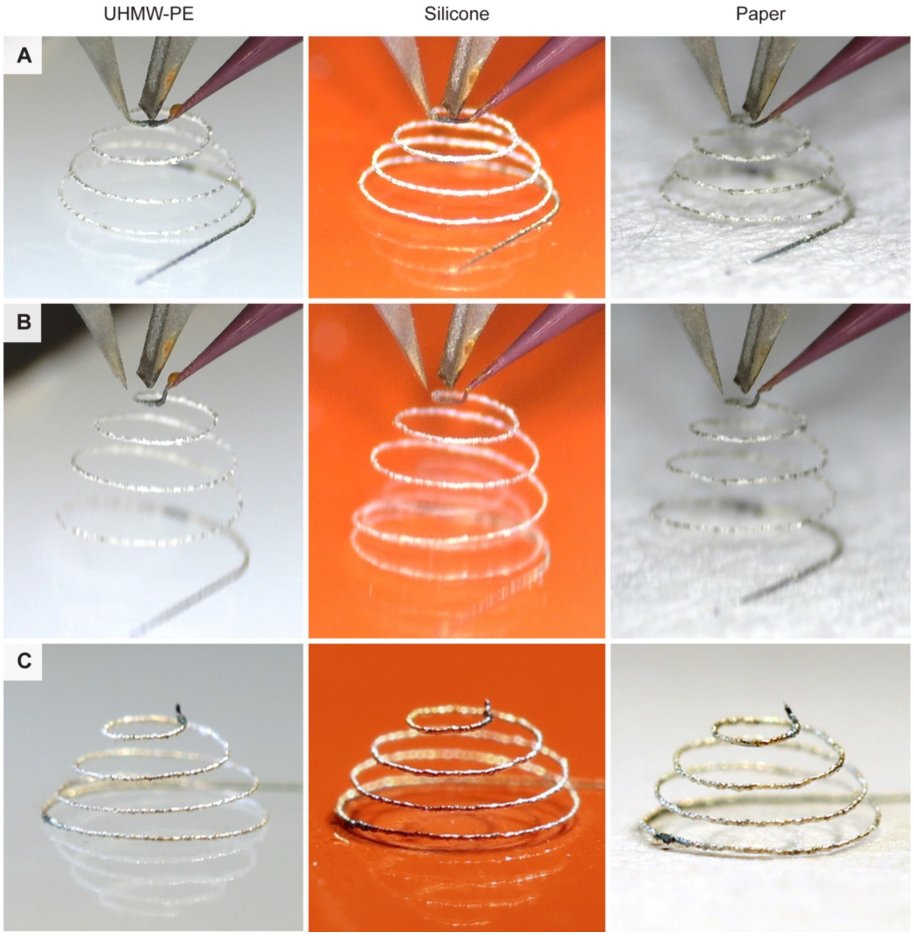

Fig. S13. Elongation of printed microspirals. Photographs show microspirals (**A**) before, (**B**) during, and (**C**) after elongation as the print nozzle is detached from the printed structure.

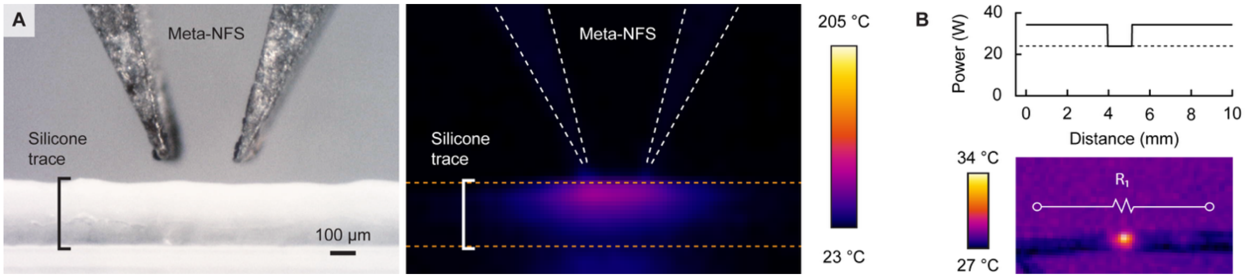

Fig. S14. Selective programming of embedded target materials. (**A**) The optical image (left) and IR image (right) show NFP heating of a silicone elastomer trace without embedded nanomaterials. (**B**) IR image showing joule heating of a silver trace programmed with a resistor (length ~600 µm) during core-shell NFP.

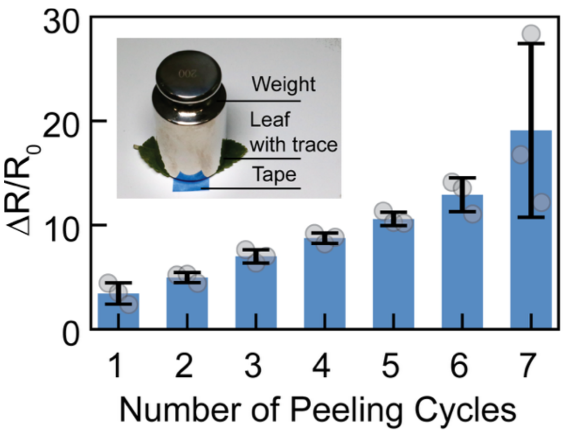

Fig. S15. Adhesion test of printed graphene traces on the leaf. A low-residual blue tape was repeatedly attached and peeled from the traces, and resistance was measured after each cycle. R_0 denotes the initial resistance of the printed graphene trace, and ΔR represents the change in resistance after peeling.

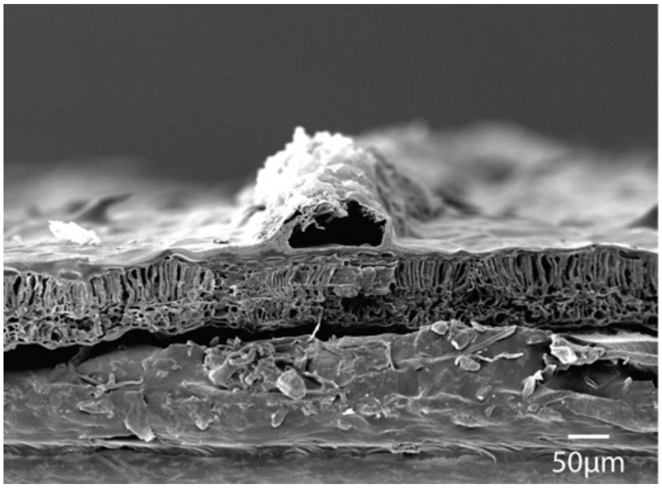

Fig. S16. Cross-sectional SEM image showing the printed graphene trace.

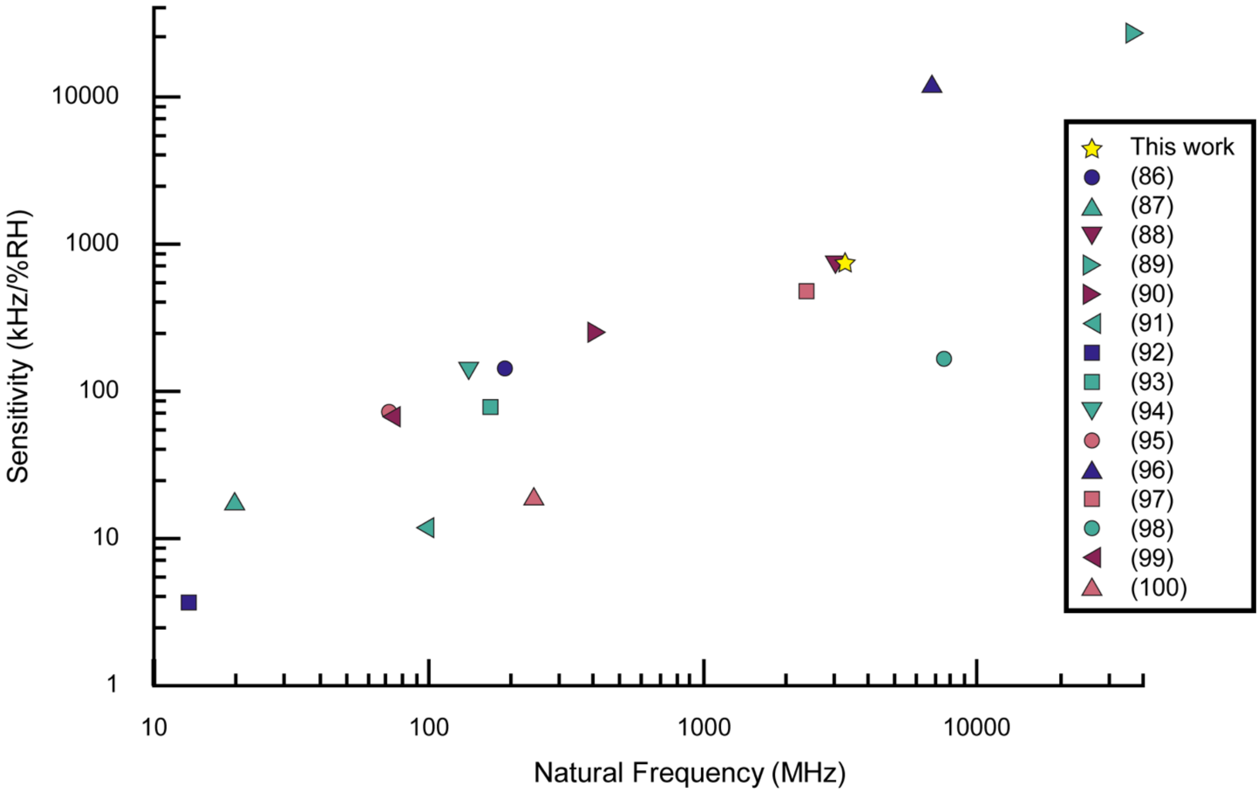

Fig. S17. Sensitivity of wireless LC humidity sensors in the literature. The wireless humidity sensor in this work has comparable sensitivity to LC wireless humidity sensors in the literature when considering its natural frequency (*86–100*).

Video S1.
3D printing of silver microarchitecture on temperature-sensitive substrates using Meta-NFS.

Video S2.
Core-shell 3D printing of a multilayer cylindrical inductor encapsulated in silicone with Meta-NFS.

Video S3.
3D printing of freestanding graphene microarchitecture on UHMW-PE with Meta-NFS even though the ink does not have the viscoelastic properties to create spanning features.

Video S4.
Meta-NFS 3D printing of a strain microsensor on a polyethylene substrate composed of an interdigitated capacitor, microantenna, and 3D interconnect.

Video S5.
Multi-material printing of planar graphene sensor and a 3D silver microantenna with Meta-NFS.

Video S6.
Multi-material printing of a humidity sensor on polyethylene with Meta-NFS.

Video S7.
A silver trace printed with Meta-NFS supporting a suspended screw.

REFERENCES

1. B. Elder, R. Neupane, E. Tokita, U. Ghosh, S. Hales, Y. L. Kong, Nanomaterial patterning in 3D printing. *Adv. Mater.* **32**, 1907142 (2020).

2. F. Han, S. Gu, A. Klimas, N. Zhao, Y. Zhao, S.-C. Chen, Three-dimensional nanofabrication via ultrafast laser patterning and kinetically regulated material assembly. *Science* **378**, 1325–1331 (2022).

3. S. Pinilla, J. Coelho, K. Li, J. Liu, V. Nicolosi, Two-dimensional material inks. *Nat. Rev. Mater.* **7**, 717–735 (2022).

4. S.-F. Liu, Z.-W. Hou, L. Lin, F. Li, Y. Zhao, X.-Z. Li, H. Zhang, H.-H. Fang, Z. Li, H.-B. Sun, 3D nanoprinting of semiconductor quantum dots by photoexcitation-induced chemical bonding. *Science* **377**, 1112–1116 (2022).

5. Y. L. Kong, I. A. Tamargo, H. Kim, B. N. Johnson, M. K. Gupta, T.-W. Koh, H.-A. Chin, D. A. Steingart, B. P. Rand, M. C. McAlpine, 3D printed quantum dot light-emitting diodes. *Nano Lett.* **14**, 7017–7023 (2014).

6. J. Bauer, C. Crook, T. Baldacchini, A sinterless, low-temperature route to 3D print nanoscale optical-grade glass. *Science* **380**, 960–966 (2023).

7. M. Zeng, Y. Du, Q. Jiang, N. Kempf, C. Wei, M. V. Bimrose, A. N. M. Tanvir, H. Xu, J. Chen, D. J. Kirsch, J. Martin, B. C. Wyatt, T. Hayashi, M. Saeidi-Javash, H. Sakaue, B. Anasori, L. Jin, M. D. McMurtrey, Y. Zhang, High-throughput printing of combinatorial materials from aerosols. *Nature* **617**, 292–298 (2023).

8. B. Y. Ahn, E. B. Duoss, M. J. Motala, X. Guo, S.-I. Park, Y. Xiong, J. Yoon, R. G. Nuzzo, J. A. Rogers, J. A. Lewis, Omnidirectional printing of flexible, stretchable, and spanning silver microelectrodes. *Science* **323**, 1590–1593 (2009).

9. J. U. Lind, T. A. Busbee, A. D. Valentine, F. S. Pasqualini, H. Yuan, M. Yadid, S.-J. Park, A. Kotikian, A. P. Nesmith, P. H. Campbell, J. J. Vlassak, J. A. Lewis, K. K. Parker, Instrumented

cardiac microphysiological devices via multimaterial three-dimensional printing. *Nat. Mater.* **16**, 303–308 (2017).

10. A. D. Valentine, T. A. Busbee, J. W. Boley, J. R. Raney, A. Chortos, A. Kotikian, J. D. Berrigan, M. F. Durstock, J. A. Lewis, Hybrid 3D printing of soft electronics. *Adv. Mater.* **29**, 1703817 (2017).

11. S. B. Walker, J. A. Lewis, Reactive silver inks for patterning high-conductivity features at mild temperatures. *J. Am. Chem. Soc.* **134**, 1419–1421 (2012).

12. M. Grouchko, A. Kamyshny, C. F. Mihailescu, D. F. Anghel, S. Magdassi, Conductive inks with a "built-in" mechanism that enables sintering at room temperature. *ACS Nano* **5**, 3354–3359 (2011).

13. M. A. Skylar-Scott, S. Gunasekaran, J. A. Lewis, Laser-assisted direct ink writing of planar and 3D metal architectures. *Proc. Natl. Acad. Sci. U.S.A.* **113**, 6137–6142 (2016).

14. Y. Zhao, J. Zhu, W. He, Y. Liu, X. Sang, R. Liu, 3D printing of unsupported multi-scale and large-span ceramic via near-infrared assisted direct ink writing. *Nat. Commun.* **14**, 2381 (2023).

15. M. S. Brown, C. B. Arnold, "Fundamentals of laser-material interaction and application to multiscale surface modification" in *Laser Precision Microfabrication* (Springer-Verlag, 2010), pp. 91–120.

16. M. Oghbaei, O. Mirzaee, Microwave versus conventional sintering: A review of fundamentals, advantages and applications. *J. Alloys Compd.* **494**, 175–189 (2010).

17. C. Leonelli, P. Veronesi, L. Denti, A. Gatto, L. Iuliano, Microwave assisted sintering of green metal parts. *J. Mater. Process. Tech.* **205**, 489–496 (2008).

18. M. Zhang, S. Fang, A. A. Zakhidov, S. B. Lee, A. E. Aliev, C. D. Williams, K. R. Atkinson, R. H. Baughman, Strong, transparent, multifunctional, carbon nanotube sheets. *Science* **309**, 1215–1219 (2005).

19. C. B. Sweeney, B. A. Lackey, M. J. Pospisil, T. C. Achee, V. K. Hicks, A. G. Moran, B. R. Teipel, M. A. Saed, M. J. Green, Welding of 3D-printed carbon nanotube–polymer composites by locally induced microwave heating. *Sci. Adv.* **3**, e1700262 (2017).

20. Z.-H. Cheng, T. Li, L. Hu, X. Ma, F. Liang, D. Zhao, B.-Z. Wang, Selectively powering multiple small-size devices spaced at diffraction limited distance with point-focused electromagnetic waves. *IEEE Trans Ind Electron* **69**, 13696–13705 (2022).

21. J. S. Ho, A. J. Yeh, E. Neofytou, S. Kim, Y. Tanabe, B. Patlolla, R. E. Beygui, A. S. Y. Poon, Wireless power transfer to deep-tissue microimplants. *Proc. Natl. Acad. Sci. U.S.A.* **111**, 7974–7979 (2014).

22. W. Li, Q. Yu, J. H. Qiu, J. Qi, Intelligent wireless power transfer via a 2-bit compact reconfigurable transmissive-metasurface-based router. *Nat. Commun.* **15**, 2807 (2024).

23. A. Imtiaz, T. M. Wallis, P. Kabos, Near-field scanning microwave microscopy: An emerging research tool for nanoscale metrology. *IEEE Microw. Mag.* **15**, 52–64 (2014).

24. K. Hall, H. Zhang, C. Furse, Design of an interstitial microwave applicator for 3D printing in the body. *IEEE J. Electromagn. RF Microw.Med. Biol.* **4**, 260–264 (2019).

25. A. Shelef, E. Jerby, Incremental solidification (toward 3D-printing) of metal powders by transistor-based microwave applicator. *Mater. Des.* **185**, 108234 (2020).

26. A. Sarmah, S. K. Desai, A. G. Crowley, G. C. Zolton, G. B. Tezel, E. M. Harkin, T. Q. Tran, K. Arole, M. J. Green, Additive manufacturing of nanotube-loaded thermosets via direct ink writing and radio-frequency heating and curing. *Carbon* **200**, 307–316 (2022).

27. A. Sarmah, S. K. Desai, G. B. Tezel, A. Vashisth, M. M. Mustafa, K. Arole, A. G. Crowley, M. J. Green, Rapid manufacturing via selective radio-frequency heating and curing of thermosetting resins. *Adv. Eng. Mater.* **24**, 2101351 (2022).

28. M. Kadic, G. W. Milton, M. van Hecke, M. Wegener, 3D metamaterials. *Nat. Rev. Phys.* **1**, 198–210 (2019).

29. R. A. Shelby, D. R. Smith, S. Schultz, Experimental verification of a negative index of refraction. *Science* **292**, 77–79 (2001).

30. S. Głowniak, B. Szczęśniak, J. Choma, M. Jaroniec, Advances in microwave synthesis of nanoporous materials. *Adv. Mater.* **33**, 2103477 (2021).

31. A. M. Schwenke, S. Hoeppener, U. S. Schubert, Synthesis and modification of carbon nanomaterials utilizing microwave heating. *Adv. Mater.* **27**, 4113–4141 (2015).

32. O. Balci, E. O. Polat, N. Kakenov, C. Kocabas, Graphene-enabled electrically switchable radar-absorbing surfaces. *Nat. Commun.* **6**, 6628 (2015).

33. C. A. Balanis, *Antenna Theory: Analysis and Design* (John Wiley & Sons, 2016).

34. M. Schnell, P. Alonso-González, L. Arzubiaga, F. Casanova, L. E. Hueso, A. Chuvilin, R. Hillenbrand, Nanofocusing of mid-infrared energy with tapered transmission lines. *Nat. Photonics* **5**, 283–287 (2011).

35. J. Hopwood, F. Iza, S. Coy, D. B. Fenner, A microfabricated atmospheric-pressure microplasma source operating in air. *J. Phys. D Appl. Phys.* **38**, 1698 (2005).

36. R. Roy, D. Agrawal, J. Cheng, S. Gedevanishvili, Full sintering of powdered-metal bodies in a microwave field. *Nature* **401**, 304–304 (1999).

37. J. Cheng, R. Roy, D. Agrawal, Radically different effects on materials by separated microwave electric and magnetic fields. *Mater. Res. Innov.* **5**, 170–177 (2002).

38. Y. Zhang, D. K. Agrawal, J. Cheng, T. Slawecki, Microwave power absorption mechanism of metallic powders. *IEEE Trans. Microw. Theory Tech.* **66**, 2107–2115 (2018).

39. D. Palessonga, M. E. Gibari, S. Ginestar, H. Terrisse, B. Guiffard, A. Kassiba, H. W. Li, Bandwidth improvement of microwave photonic components based on electro-optic polymers loaded with TiO2 nanoparticles. *Appl. Phys. A* **123**, 542 (2017).

40. P. P. Urone, R. Hinrichs, “Ch. 19. Electric potential and electric field,” in *College Physics 2e* (OpenStax, 2022), pp. 817–850.

41. M. Mehdizadeh, *Microwave/RF Applicators and Probes (Second Edition)* (William Andrew, 2015).

42. M. A. Lieberman, A. J. Lichtenberg, *Principles of Plasma Discharges and Materials Processing* (John Wiley & Sons, ed. 2, 2005).

43. Y. Sui, C. A. Zorman, R. M. Sankaran, Plasmas for additive manufacturing. *Plasma Processes Polym.* **17**, 2000009 (2020).

44. D. T. Nguyen, C. Meyers, T. D. Yee, N. A. Dudukovic, J. F. Destino, C. Zhu, E. B. Duoss, T. F. Baumann, T. Suratwala, J. E. Smay, R. Dylla-Spears, 3D-printed transparent glass. *Adv. Mater.* **29**, 1701181 (2017).

45. R. M. Cywar, N. A. Rorrer, C. B. Hoyt, G. T. Beckham, E. Y.-X. Chen, Bio-based polymers with performance-advantaged properties. *Nat. Rev. Mater.* **7**, 83–103 (2022).

46. M. Li, A. Pal, A. Aghakhani, A. Pena-Francesch, M. Sitti, Soft actuators for real-world applications. *Nat. Rev. Mater.* **7**, 235–249 (2022).

47. P. Wang, M. Hu, H. Wang, Z. Chen, Y. Feng, J. Wang, W. Ling, Y. Huang, The evolution of flexible electronics: From nature, beyond nature, and to nature. *Adv. Sci.* **7**, 2001116 (2020).

48. N. A. Patil, J. Njuguna, B. Kandasubramanian, UHMWPE for biomedical applications: Performance and functionalization. *Eur. Polym. J.* **125**, 109529 (2020).

49. Z. Hui, L. Zhang, G. Ren, G. Sun, H. Yu, W. Huang, Green flexible electronics: Natural materials, fabrication, and applications. *Adv. Mater.* **35**, 2211202 (2023).

50. I. D. Robertson, M. Yourdkhani, P. J. Centellas, J. E. Aw, D. G. Ivanoff, E. Goli, E. M. Lloyd, L. M. Dean, N. R. Sottos, P. H. Geubelle, J. S. Moore, S. R. White, Rapid energy-efficient

manufacturing of polymers and composites via frontal polymerization. *Nature* **557**, 223–227 (2018).

51. M. Chen, Z. Zhou, S. Hu, N. Huang, H. Lee, Y. Liu, J. Yang, X. Huan, Z. Xu, S. Cao, X. Cheng, T. Wang, S. F. Yu, B. P. Chan, J. Tang, S. Feng, J. T. Kim, 3D printing of arbitrary perovskite nanowire heterostructures. *Adv. Funct. Mater.* **33**, 2212146 (2023).

52. Y. Park, I. Yun, W. G. Chung, W. Park, D. H. Lee, J. Park, High-resolution 3D printing for electronics. *Adv. Sci.* **9**, 2104623 (2022).

53. J. J. Adams, S. C. Slimmer, J. A. Lewis, J. T. Bernhard, 3D-printed spherical dipole antenna integrated on small RF node. *Electron. Lett.* **51**, 661–662 (2015).

54. C. Pfeiffer, X. Xu, S. R. Forrest, A. Grbic, Direct transfer patterning of electrically small antennas onto three-dimensionally contoured substrates. *Adv. Mater.* **24**, 1166–1170 (2012).

55. P. K. Sharma, N. Gupta, P. I. Dankov, Characterization of polydimethylsiloxane (PDMS) as a wearable antenna substrate using resonance and planar structure methods. *AEU - Int. J. Electron. Commun.* **127**, 153455 (2020).

56. R. Rayhana, G. G. Xiao, Z. Liu, Printed sensor technologies for monitoring applications in smart farming: A review. *IEEE Trans. Instrum. Meas.* **70**, 1–19 (2021).

57. T. T. S. Lew, V. B. Koman, P. Gordiichuk, M. Park, M. S. Strano, The emergence of plant nanobionics and living plants as technology. *Adv. Mater. Technol.* **5**, 1900657 (2020).

58. J. P. Giraldo, H. Wu, G. M. Newkirk, S. Kruss, Nanobiotechnology approaches for engineering smart plant sensors. *Nat. Nanotechnol.* **14**, 541–553 (2019).

59. Y. Lu, K. Xu, L. Zhang, M. Deguchi, H. Shishido, T. Arie, R. Pan, A. Hayashi, L. Shen, S. Akita, K. Takei, Multimodal plant healthcare flexible sensor system. *ACS Nano* **14**, 10966–10975 (2020).

60. J. M. Nassar, S. M. Khan, D. R. Villalva, M. M. Nour, A. S. Almuslem, M. M. Hussain, Compliant plant wearables for localized microclimate and plant growth monitoring. *NPJ Flexible Electron.* **2**, 24 (2018).

61. S. Yin, H. Ibrahim, P. S. Schnable, M. J. Castellano, L. Dong, A field-deployable, wearable leaf sensor for continuous monitoring of vapor-pressure deficit. *Adv. Mater. Technol.* **6**, 2001246 (2021).

62. S. Wang, Y. Diao, Printed electronics for cultivating plants in space. *Nat. Rev. Mater.* **9**, 762–763 (2024).

63. E. Vázquez, M. Prato, Carbon nanotubes and microwaves: Interactions, responses, and applications. *ACS Nano* **3**, 3819–3824 (2009).

64. J. P. Mensing, T. Lomas, A. Tuantranont, 2D and 3D printing for graphene based supercapacitors and batteries: A review. *Sustain. Mater. Technol.* **25**, e00190 (2020).

65. M. Chen, X. Qin, G. Zeng, Biodegradation of carbon nanotubes, graphene, and their derivatives. *Trends Biotechnol.* **35**, 836–846 (2017).

66. E. Jerby, Y. Meir, A. Salzberg, E. Aharoni, A. Levy, J. P. Torralba, B. Cavallini, Incremental metal-powder solidification by localized microwave-heating and its potential for additive manufacturing. *Addit. Manuf.* **6**, 53–66 (2015).

67. X. Qi, J. Xu, Q. Hu, Y. Deng, R. Xie, Y. Jiang, W. Zhong, Y. Du, Metal-free carbon nanotubes: Synthesis, and enhanced intrinsic microwave absorption properties. *Sci. Rep.* **6**, 28310 (2016).

68. J. Liu, Z. Jia, W. Zhou, X. Liu, C. Zhang, B. Xu, G. Wu, Self-assembled MoS_2/magnetic ferrite $CuFe_2O_4$ nanocomposite for high-efficiency microwave absorption. *Chem. Eng. J.* **429**, 132253 (2022).

69. M. Green, P. Xiang, Z. Liu, J. Murowchick, X. Tan, F. Huang, X. Chen, Microwave absorption of aluminum/hydrogen treated titanium dioxide nanoparticles. *J. Mater.* **5**, 133–146 (2019).

70. H. Bayrakdar, Complex permittivity, complex permeability and microwave absorption properties of ferrite–paraffin polymer composites. *J. Magn. Magn. Mater.* **323**, 1882–1885 (2011).

71. Z. Dong, M. Vuckovac, W. Cui, Q. Zhou, R. H. A. Ras, P. A. Levkin, 3D printing of superhydrophobic objects with bulk nanostructure. *Adv. Mater.* **33**, 2106068 (2021).

72. H. Wang, Q. Ruan, H. Wang, S. D. Rezaei, K. T. P. Lim, H. Liu, W. Zhang, J. Trisno, J. Y. E. Chan, J. K. W. Yang, Full color and grayscale painting with 3D printed low-index nanopillars. *Nano Lett.* **21**, 4721–4729 (2021).

73. M. Ali, F. Alam, N. Vahdati, H. Butt, 3D-printed holographic fresnel lenses. *Adv. Eng. Mater.* **24**, 2101641 (2022).

74. Y. Mu, K. Sun, Y. Jia, N. Zhang, S. Wu, Y. Jia, G. Wang, 3D-printed strong and ductile high-entropy alloys with orientation arranged nanostructure complex. *J. Alloys Compd.* **968**, 171824 (2023).

75. B. Weidinger, G. Yang, N. von Coelln, H. Nirschl, I. Wacker, P. Tegeder, R. R. Schröder, E. Blasco, 3D printing hierarchically nano-ordered structures. *Adv. Sci.* **10**, 2302756 (2023).

76. A. Albrecht, A. Rivadeneyra, A. Abdellah, P. Lugli, J. F. Salmerón, Inkjet printing and photonic sintering of silver and copper oxide nanoparticles for ultra-low-cost conductive patterns. *J. Mater. Chem. C* **4**, 3546–3554 (2016).

77. Y.-T. Kwon, Y.-S. Kim, Y. Lee, S. Kwon, M. Lim, Y. Song, Y.-H. Choa, W.-H. Yeo, Ultrahigh conductivity and superior interfacial adhesion of a nanostructured, photonic-sintered copper membrane for printed flexible hybrid electronics. *ACS Appl. Mater. Interfaces* **10**, 44071–44079 (2018).

78. S. Majee, W. Zhao, A. Sugunan, T. Gillgren, J. A. Larsson, R. Brooke, N. Nordgren, Z. Zhang, S. Zhang, D. Nilsson, A. Ahniyaz, Highly conductive films by rapid photonic annealing of inkjet printable starch–graphene ink. *Adv. Mater. Interfaces* **9**, 2101884 (2022).

79. Y. Kim, H. Yuk, R. Zhao, S. A. Chester, X. Zhao, Printing ferromagnetic domains for untethered fast-transforming soft materials. *Nature* **558**, 274–279 (2018).

80. F. Kotz, K. Arnold, W. Bauer, D. Schild, N. Keller, K. Sachsenheimer, T. M. Nargang, C. Richter, D. Helmer, B. E. Rapp, Three-dimensional printing of transparent fused silica glass. *Nature* **544**, 337–339 (2017).

81. M. S. Saleh, C. Hu, R. Panat, Three-dimensional microarchitected materials and devices using nanoparticle assembly by pointwise spatial printing. *Sci. Adv.* **3**, e1601986 (2017).

82. T. J. K. Buchner, S. Rogler, S. Weirich, Y. Armati, B. G. Cangan, J. Ramos, S. T. Twiddy, D. M. Marini, A. Weber, D. Chen, G. Ellson, J. Jacob, W. Zengerle, D. Katalichenko, C. Keny, W. Matusik, R. K. Katzschmann, Vision-controlled jetting for composite systems and robots. *Nature* **623**, 522–530 (2023).

83. M. A. Skylar-Scott, J. Mueller, C. W. Visser, J. A. Lewis, Voxelated soft matter via multimaterial multinozzle 3D printing. *Nature* **575**, 330–335 (2019).

84. W. Jung, Y.-H. Jung, P. V. Pikhitsa, J. Feng, Y. Yang, M. Kim, H.-Y. Tsai, T. Tanaka, J. Shin, K.-Y. Kim, H. Choi, J. Rho, M. Choi, Three-dimensional nanoprinting via charged aerosol jets. *Nature* **592**, 54–59 (2021).

85. S. Ling, X. Tian, Q. Zeng, Z. Qin, S. A. Kurt, Y. J. Tan, J. Y. H. Fuh, Z. Liu, M. D. Dickey, J. S. Ho, B. C. K. Tee, Tension-driven three-dimensional printing of free-standing field's metal structures. *Nat. Electron.* **7**, 671–683 (2024).

86. M.-Z. Xie, L.-F. Wang, L. Dong, W.-J. Deng, Q.-A. Huang, Low cost paper-based LC wireless humidity sensors and distance-insensitive readout system. *IEEE Sensors J.* **19**, 4717–4725 (2019).

87. Q.-Y. Ren, L.-F. Wang, J.-Q. Huang, C. Zhang, Q.-A. Huang, Simultaneous remote sensing of temperature and humidity by LC-type passive wireless sensors. *J. Microelectromech. Syst.* **24**, 1117–1123 (2015).

88. M. Borgese, F. A. Dicandia, F. Costa, S. Genovesi, G. Manara, An inkjet printed chipless RFID sensor for wireless humidity monitoring. *IEEE Sensors J.* **17**, 4699–4707 (2017).

89. X. Lin, B.-C. Seet, F. Joseph, Wearable humidity sensing antenna for BAN applications over 5G networks. *2018 IEEE 19th Wirel. Microw. Technol. Conf. (WAMICON)* (2018); pp. 1–4.

90. W. Lv, Y. Zhang, H. Luo, Q. Xu, W. Quan, J. Yang, M. Zeng, N. Hu, Z. Yang, Wide remote-range and accurate wireless LC temperature–humidity sensor enabled by efficient mutual interference mitigation. *ACS Sens.* **8**, 4531–4541 (2023).

91. Y. Li, Z. Wei, J. Huang, An LC-type flexible wireless humidity sensor with electrospun isolation layer. *2021 IEEE Sens.* **00**, 1–4 (2021).

92. Y.-B. Xue, H.-Y. Huang, W.-J. Zhu, B.-J. Chen, Y.-J. Ju, C. Feng, Wireless, passive paper-based LC humidity sensor with RFID frequency of 13.56 MHz. *IEEE Sensors J.* **24**, 15800–15810 (2024).

93. S. Su, W. Lv, T. Zhang, Q. Tan, W. Zhang, J. Xiong, A MoS_2 nanoflakes-based LC wireless passive humidity sensor. *Sensors* **18**, 4466 (2018).

94. W.-J. Deng, L.-F. Wang, L. Dong, Q.-A. Huang, Experimental study of the bending effect on LC wireless humidity sensors fabricated on flexible PET substrates. *J. Microelectromech. Syst.* **27**, 761–763 (2018).

95. W. Lv, Q. Tan, H. Kou, W. Zhang, J. Xiong, MWCNTs/WS_2 nanocomposite sensor realized by LC wireless method for humidity monitoring. *Sens. Actuators A: Phys.* **290**, 207–214 (2019).

96. E. M. Amin, M. S. Bhuiyan, N. C. Karmakar, B. Winther-Jensen, Development of a low cost printable chipless RFID humidity sensor. *IEEE Sensors J.* **14**, 140–149 (2013).

97. J.-K. Park, T.-G. Kang, B.-H. Kim, H.-J. Lee, H. H. Choi, J.-G. Yook, Real-time humidity sensor based on microwave resonator coupled with PEDOT:PSS conducting polymer film. *Sci. Rep.* **8**, 439 (2018).

98. T.-K. Nguyen, C.-H. Tseng, A new microwave humidity sensor with near-field self-injection-locked technology. *IEEE Sensors J.* **21**, 21520–21528 (2021).

99. C. Zhang, L. Guo, L. Wang, J. Huang, Q. Huang, Passive wireless integrated humidity sensor based on dual-layer spiral inductors. *Electron. Lett.* **50**, 1287–1289 (2014).

100. C. Zhang, L.-F. Wang, J.-Q. Huang, Q.-A. Huang, An LC-type passive wireless humidity sensor system with portable telemetry unit. *J. Microelectromech. Syst.* **24**, 575–581 (2015).
